# Single cell atlas decodes the molecular dynamics of scar repair after human rotator cuff tear

**DOI:** 10.1038/s41413-025-00501-5

**Published:** 2026-02-05

**Authors:** Yiming Qin, Guang Yang, Tao Zhang, Yuying Yang, Liyang Wan, Tao Zhang, Linfeng Wang, Zhiyu Hu, Zhu Dai, Hongkang Zhou, Chengjun Li, Jianzhong Hu, Hongbin Lu

**Affiliations:** 1 Department of Sports Medicine, Xiangya Hospital, Central South University, Changsha, China; 2 Department of Spine Surgery and Orthopaedics, Xiangya Hospital, Central South University, Changsha, China; 3 Key Laboratory of Organ Injury, Aging and Regenerative Medicine of Hunan Province, Changsha, China; 4 Mobile Health Ministry of Education, China Mobile Joint Laboratory, Changsha, China; 5 National Clinical Research Center for Geriatric Disorders, Xiangya Hospital, Central South University, Changsha, China; 6 Department of Rehabilitation Medicine, Union Hospital, Tongji Medical College of Huazhong University of Science and Technology, Wuhan, China; 7 Hunan Engineering Research Center of Sports and Health, Changsha, China; 8 Department of Orthopedics, The Fifth Affiliated Hospital, Sun Yat-sen University, Zhuhai, China; 9 Institute for Advanced Study, Central South University, Changsha, China

**Keywords:** Bone, Pathogenesis

## Abstract

Irreversible fibrotic scarring after rotator cuff tear (RCT) compromises the mechanical properties of the healing tendon, yet the underlying mechanisms remain poorly understood. Here, we analyzed the histological features of human RCT scars, characterized by disruption of tendon architecture, disorganized collagen fibrils, and imbalance in type I/III collagen ratios and fibril diameters. Using single-cell RNA sequencing of tendon stumps from patients with RCT, we deconvolved the cellular and molecular landscape of the fibrotic scarring microenvironment. Heterogenous pro-fibrotic subclusters were identified and validated to participate into scar formation, including tendon stem cell, senescent tenocyte, SOX9-driven pro-fibrotic macrophage, and pro-fibrotic endothelial cells undergoing endothelial-mesenchymal transition (EndoMT). Furthermore, we found that osteopontin and TGF-β signaling were key drivers of extracellular matrix deposition, and their blockade ameliorated fibrotic scarring after RCT. Collectively, our study dissected the dynamic scarring microenvironment in human RCT and highlights potential therapeutic targets for preventing pathological scar formation.

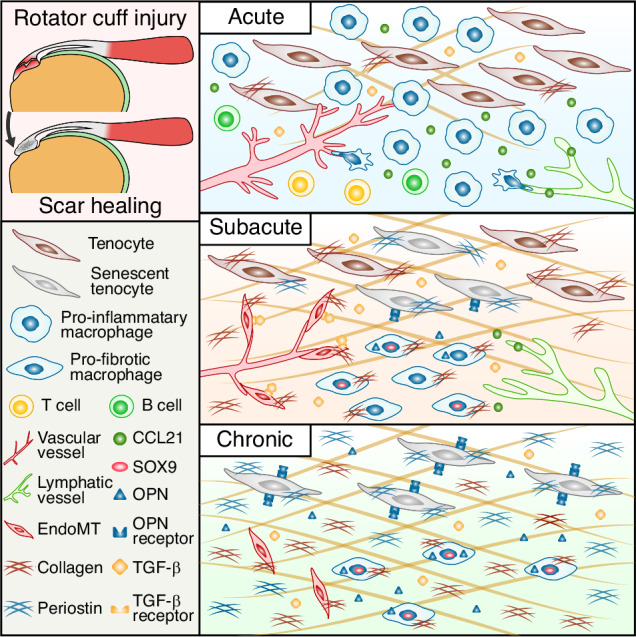

## Introduction

Scar healing is a common outcome of tendon repair after rotator cuff tear (RCT),^1–4^ which alters tendon architecture and mechanical properties,^5,6^ increasing disability and risk of re-tear after surgery.^7,8^ Fibrotic scar results from inflammation, impaired regeneration, and activation of pro-fibrotic signaling, causing disrupted extracellular matrix (ECM) accumulation.^9^ Heterogeneous pathological microenvironment contributes to fibrotic scar formation after rotator cuff injury.

Predominantly, fibrotic scarring is initiated by inflammation. Different inflammatory populations, such as macrophages, neutrophils, lymphocytes, cytokines, chemokines and necrotic debris jointly activate the inflammatory response after the rotator cuff injury.^10–12^ Inflammatory microenvironment acts as a double-edged sword: on one hand, it damages tissue, while on the other hand, it promotes tissue repair. Current studies reveal immune cells, especially macrophages, are the major source of secreted transforming growth factor-β (TGF-β), which promoting tissue repair and fibrosis.^13–16^ During inflammation subsiding, macrophages transformed into a profibrotic phenotype of scar-associated macrophages (SAMs), which highly express CD9, TREM2, SPP1, COL1A1 and other scar-related genes,^17–19^ contributing to scar formation in diverse fibrotic disease.^11,20,21^ However, the mechanisms of inflammatory components participating into fibrotic scar after rotator cuff injury remain unclear.

Impairment of regenerative ability contribute to fibrotic scarring.^6^ During RCT repair, newly synthesized ECM components, composed mainly of type I and type III collagen,^22^ deposited chaotically and lost normal organization of tendon fibers, which matures into a scar^1–3^ and needs remodeling to restore order.^23^ Tissue remodeling depends on balanced ECM anabolic and catabolic ability.^6,23,24^ Homeostatic ECM synthesis maintains ECM volume, whereas matrix metalloproteinases (MMPs), such as MMP-1, MMP-2 and MMP-13, cleaves collagen fibrils.^23,25^ Fetal tendon injury heals scarlessly characterized by restoration of normal tendon architecture, which might stem from its intrinsic regenerative ability.^26–28^ However, intrinsic remodeling ability of adult tendon is limited,^29^ which causing fibrotic scar never be fully replaced.^1–3^ The mechanisms of limited remodeling ability of human adult tendon need to be further explored.

Multiple pro-fibrotic signals contribute to fibrotic scarring, such as TGF-β,^13,30^ Osteopontin (OPN),^31,32^ bone morphogenetic protein (BMP)^33^ and platelet­derived growth factor BB (PDGF-BB).^34^ TGF-β signaling is activated in various fibrosis disease.^13^ Potential mechanisms of TGF-β signaling in fibrosis include induction of myofibroblasts phenoconversion,^35^ macrophages secretion of pro-fibrotic factors^36^ and endothelial-to-mesenchymal transition (EndoMT).^37,38^ OPN, encoded by SPP1 gene,^39,40^ is a macrophage-derived pro-fibrotic cytokine^41,42^ promoting ECM deposition by binding to integrin receptors of different cell types.^43^ However, activation characteristics and mechanisms of diverse pro-fibrotic signals in RCT are unknown.

To develop a comprehensive understanding of dynamic fibrotic scar characteristics of RCT, we characterized histological features of RCT scarring and performed single-cell RNA sequencing of 9 tendon stumps from different stages of human RCT. We identified multiple pro-fibrotic phenotypes of tenocytes, macrophages and endothelial cells, including tendon stem cells (TSCs), senescent tenocytes, pro-fibrotic macrophages, pro-fibrotic endothelial cells. We further found several key transcription factors, with DBP and FOXO1 driving tenocyte senescence and SOX9 driving a pro-fibrotic macrophage phenotype. We also profiled diverse pro-fibrotic signaling across distinct cell subclusters of RCT. Together, dynamic characteristics of diverse pro-fibrotic factors contributed to scar formation in human RCT, which provided potential insights of targets for reversing RCT scarring.

## Results

### Fibrotic scar formation after human RCT

**Fig. 1:**
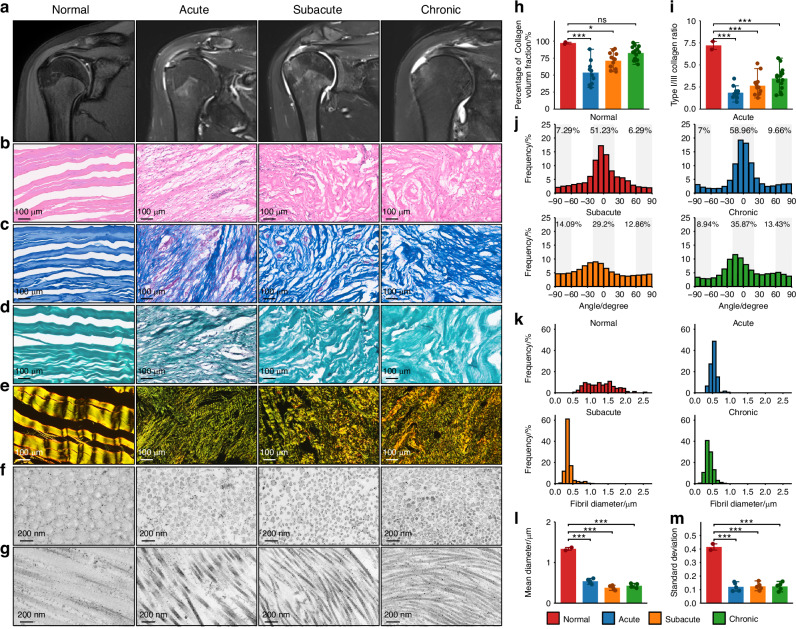
Scar formation after human rotator cuff injury. **a** MRI imaging of normal and different stages (acute, subacute, and chronic) of human RCT. Histological imaging of H&E (**b**), Masson (**c**), Safranin O-Fast Green (**d**), polarized light images of picrosirius red staining (**e**), and electron microscopy of collagen fibrils (**f**, **g**), in different stages of human RCT. **h**, **i** Bar plots showing collagen volume fraction and type I/III collagen ratio of normal tendon and different stages of human RCT (Normal, *n* = 2; Acute, *n* = 10; Subacute, *n* = 13; Chronic, *n* = 15). **j**, **k** Collagen fiber angle and collagen fibril diameter distribution of normal tendon and different stages of human RCT. **l**, **m** Bar plots showing mean value and standard deviation of collagen fibril diameters for normal tendon and different stages of human RCT (Normal, *n* = 2; Acute, *n* = 5; Subacute, *n* = 5; Chronic, *n* = 5). (mean ± SD are shown as error bar, *P < 0.05, **P < 0.01, ***P < 0.001)

To investigate the histological characteristics of scar healing after RCT, we collected tendon tissues from 10, 13, and 15 cases of acute, subacute, and chronic RCT, and 2 normal semitendinosus tendon tissues as normal controls. MRI showed obvious injury signs for different stages of RCT (Fig. 1a). H&E, Masson, Safranin O-Fast Green, picrosirius red staining and transmission electron microscopy imaging were performed on tissues of different stages (Fig. 1b–g). H&E, Masson and Safranin O-Fast Green staining revealed that normal tendon collagen fascicles were densely arranged in parallel with sparse embedded cells. During the acute stage of RCT, collagen fascicles appeared sparse and disorganized, accompanied by massive cell infiltration, extensive neovascularization (Fig. 1b–g), and significant reduction in collagen volume fraction (CVF) (Fig. 1h). In subacute and chronic stages, cell infiltration diminished, blood vessels gradually regressed, and collagen fascicles became denser but more disordered (Fig. 1b–d), with CVF increasing but remaining below normal fraction (Fig. 1h). Picrosirius red staining revealed that normal tendon predominantly comprised type I collagen, with a type I/III collagen ratio of approximately 7. Following RCT, type III collagen deposition increased, leading to a significant and persistent reduction in the type I/III collagen ratio, which persisted into the chronic stage (Fig. 1i). Fiber angle analysis also revealed that disordered arrangement of collagen fascicles persisted post injury (Fig. 1j). Transmission electron microscopy (TEM) demonstrated that diameters of normal collagen fibrils were uniformly distributed within the range of 1–2 μm. In contrast, collagen fibrils of different stages of RCT exhibited a significant reduction in diameter, with a peak around 0.5 μm and a smaller standard deviation (Fig. 1k–m). Similarly, TEM confirmed disordered arrangement of collagen fibrils post injury (Fig. 1f, g). To investigate the cross-species characteristics of scar healing in RCT, we established rat model of supraspinatus tendon rupture (Fig. S1a). Histological analysis using H&E and Masson staining revealed that the scar formation process in rat model was similar to the temporal progression observed in human RCT (Fig. S1b). These findings indicate that RCT repair result in persistent scar formation, characterized by disordered tissue architecture, chaos of collagen arrangement, imbalanced type I/III collagen ratio and thinner collagen fibril diameters.

### Single cell atlas and fibrotic microenvironment profile of RCT

**Fig. 2:**
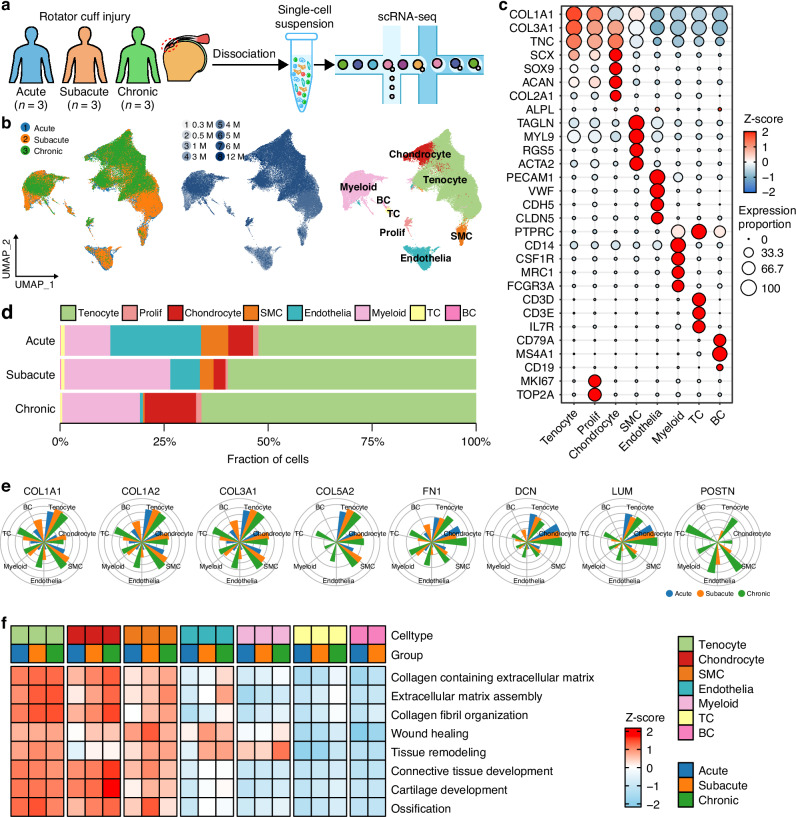
Fibrotic scar profile across stages and cell types after RCT. **a** Schematic of workflow for scRNA-seq. **b** UMAP embedding of 87 730 cells from 9 RCT samples annotated by disease stages, injury duration and cell types. **c** Dot heatmap showing common marker genes expression of each major cell type. **d** Proportion of major cell types in different RCT stages. **e** FRGs expression in different RCT stages for each cell type. **f** ECM secretion and fibrosis signaling related pathways activity in different RCT stages for each cell type

To investigate the microenvironment characteristics of fibrotic scar healing after RCT, we performed single-cell RNA sequencing for tendon stumps of acute, subacute and chronic stages of adult human RCT (Fig. 2a). After quality control and integration, we obtained a total of 87 730 high quality cells, which were identified as eight major cell types, including tenocyte, chondrocyte, smooth muscle cell (SMC), endothelial cell (endothelia), proliferative cell (prolif), myeloid cell (myeloid), B cell (BC) and T cell (TC), based on canonical marker genes (Fig. 2b, c, Fig. S1c–e and Table S2). Tenocyte was the dominant cell type across different stages. Myeloid proportion increased in subacute and chronic stages, while endothelial and SMC proportions gradually regressed as the duration of RCT progressed (Fig. 2d and Fig. S1f).

Subsequently, we focused on fibrotic scarring profile across different cell types in RCT. We curated 8 fibrosis-related genes (FRGs), including COL1A1, COL1A2, COL3A1, COL5A2, FN1, DCN, LUM, and POSTN, encoding ECM proteins closely related to fibrosis (Table S3). FRGs were highly expressed in tenocyte, chondrocyte, and SMC across all stages. Importantly, FRGs expression was significantly increased in other cell types, including myeloid cell, endothelial cell and TC, particularly during the subacute and chronic stages (Fig. 2e), indicating their potential contribution to scar healing. Among them, POSTN, encoding periostin which drives fibrosis progression across multiple diseases,^44–46^ showed exclusively upregulation at the chronic stage across most cell types (Fig. 2e). Consistently, ECM related pathways were significantly enriched in tenocyte, chondrocyte and SMC (Fig. 2f). Together, these findings indicate that fibrotic healing after RCT involves not only structural cells but also immune and vascular cell populations.

### Tendon stem cells involved in fibrotic scarring after RCT

**Fig. 3:**
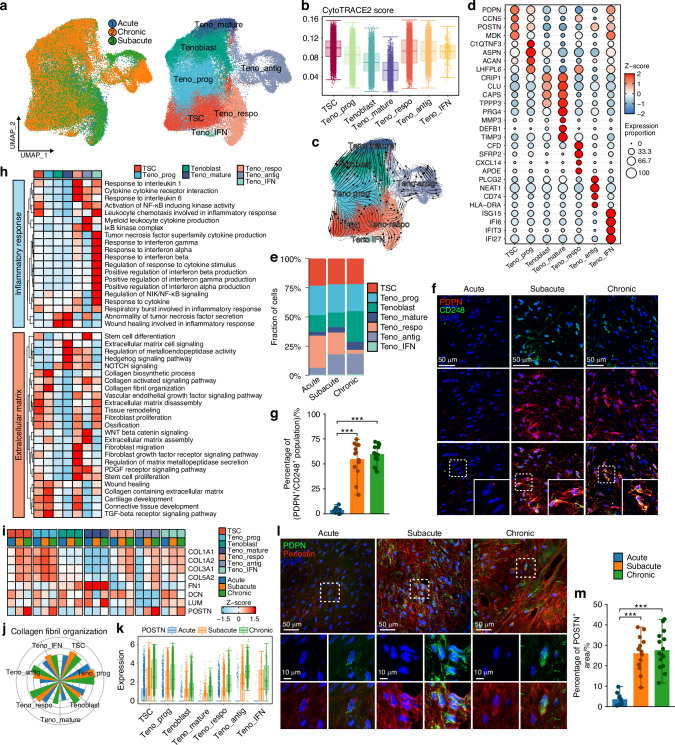
Tendon stem cell and tenocyte progenitor as the main source of ECM after RCT. **a** UMAP embedding of tendon cells annotated by stages and subclusters. **b** Proportion of tenocyte subclusters in different RCT stages. **c** Dot heatmap showing significant upregulated genes of each subcluster. **d** Heatmap of inflammation and ECM related pathways activities in tenocyte subclusters. **e** Violin plot of CytoTRACE2 scores in tenocyte subclusters. **f** Steady-state RNA velocity of tenocyte subclusters. **g** Immunofluorescence of stem-related genes (PDPN and CD248) labeling tendon stem cells in different stages. **h** Measurement of proportion of PDPN^+^/CD248^+^ cells in RCT (Acute, *n* = 10; Subacute, *n* = 13; Chronic, *n* = 15). **i** Heatmap of FRGs expression in tenocyte subclusters. **j** Pathway activity of “collagen fibril assembly” in different stages of each subcluster. **k** Boxplot showing POSTN expression in different stages of each subcluster. **l** Immunofluorescence labeling PDPN and periostin expression in RCT. **m** Measurement of POSTN^+^ area in RCT (Acute, *n* = 10; Subacute, *n* = 13; Chronic, *n* = 15). (mean ± SD are shown as error bar, *P < 0.05, **P < 0.01, ***P < 0.001)

We performed re-clustering on tenocytes and identified seven subclusters based on pathway enrichment and cell stemness scores. Four represented common developmental states: tendon stem cell (TSC), tenocyte progenitor (Teno_prog), Tenoblast, and mature tenocyte (Teno_mature), with a progressive decline in stemness and increase in maturity from TSC to Teno_mature (Fig. 3a–c). Additionally, three functionally specialized subclusters were identified: interferon-related tenocyte (Teno_IFN), antigen presentation-related tenocyte (Teno_antig), and response-related tenocyte (Teno_respo, enriched in “response to extracellular stimulus”-related pathways) (Fig. 3a, Tables S4, S5). Top differentially upregulated genes of each subcluster were displayed (Fig. 3d). Along RCT progression, the proportion of TSC remained unchanged, and Teno_mature did not increased as expected. In contrast, the proportion of Tenoblast increased at chronic stage (Fig. 3e). Immunofluorescence further confirmed the significant presence of tendon stem/progenitor cells during subacute and chronic stages (Fig. 3f, g). These indicated that TSC and Teno_progdid not differentiate into mature tenocytes gradually after RCT. Functional analysis revealed that inflammation-related pathways were significantly activated in Teno_IFN, Teno_respo and Teno_antig (Fig. 3h), indicating their roles in inflammation response. Conversely, ECM-related pathways and FRGs were significantly upregulated in TSC and Teno_prog (Fig. 3h–j), suggesting their potential roles driving fibrotic healing. Notably, POSTN predominantly expressed in TSC and Teno_prog and exhibited stage-dependent upregulation as RCT progressed (Fig. 3i, k). Immunofluorescence further demonstrated marked periostin deposition during subacute and chronic stages (Fig. 3l, m). Altogether, these results indicate that tenocyte subclusters with higher stemness, particularly TSC and Teno_prog, are sustained throughout the subacute and chronic stages, exhibit high expression of fibrosis-related ECM proteins (such as periostin), and are involved in scar healing process after RCT.

### Senescence impaired tissue remodeling ability of tenocyte

**Fig. 4:**
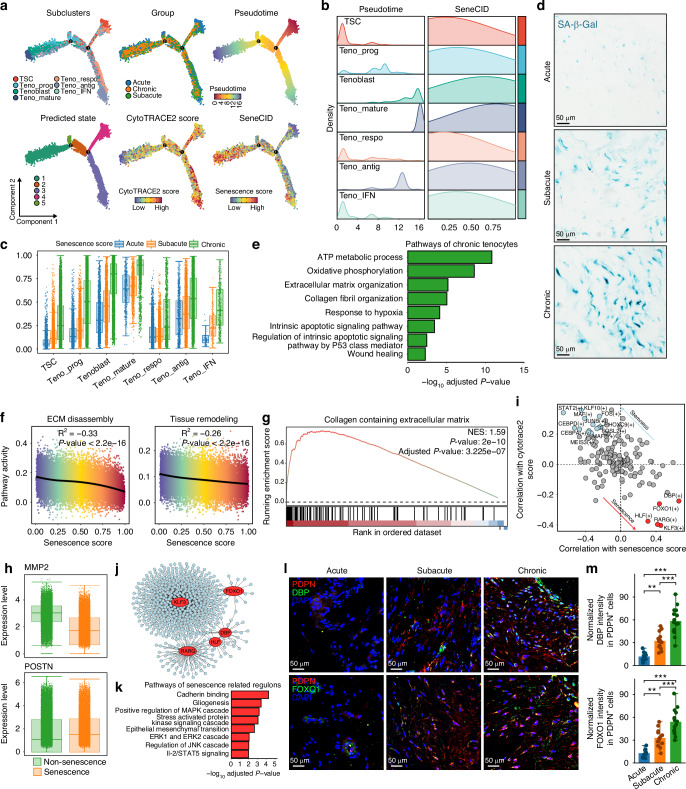
Characteristics and key regulators of senescent tenocyte after RCT. **a** Tenocyte development trajectories annotated by pseudotime, predicted state, RCT stage, subclusters, CytoTRACE2 and senescence scores. **b** Ridge plots showing pseudotime and senescence score across tenocyte subclusters. **c** Boxplot showing senescence scores in different stages of each tenocyte subcluster. **d** SA-β-Gal staining of different stages. **e** Pathways enriched in tenocytes of chronic stage. **f** Correlation between senescence score and “extracellular matrix disassembly” as well as “tissue remodeling” pathways in tenocytes. **g** GSEA plot of pathway “Collagen Containing Extracellular Matrix” in senescent versus non-senescent tenocytes. **h** Boxplot showing expression of MMP2 and POSTN in senescent and non-senescent tenocytes. **i** Correlation analysis between regulons and CytoTRACE2 as well as senescence scores. **j** Regulatory networks of senescence-driven regulons. **k** Pathways enriched in senescence-driven regulons. **l** Immunofluorescence showing DBP and FOXO1 expression of tendon stem cells of RCT. **m** Measurement of DBP and FOXO1 intensities in PDPN^+^ cells (Acute, *n* = 10; Subacute, *n* = 13; Chronic, *n* = 15). (mean ± SD are shown as error bar, *P < 0.05, **P < 0.01, ***P < 0.001)

Senescence plays a crucial role in promoting fibrosis.^47–49^ To explore tenocyte senescence during differentiation across different stages of RCT, we conducted senescence evaluation using SenCID and trajectory analysis based on Monocle. Two differentiation trajectories were identified (Fig. 4a). Trajectory-1, originating from TSC, progressed through Teno_IFN, Teno_respo, and Teno_antig, maintaining consistently high CytoTRACE and low senescence scores. Trajectory-2 represented the maturation process of tenocyte, progressing from TSC to Teno_prog, then to Tenoblast, and ultimately to teno_mature, with a significant increase of senescence scores, where Teno_mature exhibited the highest senescence score (Fig. 4a–c and Fig. S2a). Moreover, as RCT progressed, the senescence scores of all tenocyte subclusters significantly increased (Fig. 4c and Fig. S2b), suggesting that tenocytes underwent progressive senescence after RCT. SA-β-Gal staining further confirmed this finding (Fig. 4d). Enrichment analysis revealed that tenocytes significantly enriched in pathways related to cellular respiration, hypoxia, apoptosis, and P53 signaling in the chronic stage (Fig. 4e), which may contribute to tenocyte senescence.

We next explored whether tenocyte senescence affects the remodeling of scar tissue, a critical process in fibrosis resolution.^50–52^ We found that tenocyte senescence scores were negatively correlated with “extracellular matrix disassembly” and “tissue remodeling” pathways (Fig. 4f). In contrast, GSEA analysis revealed that senescent tenocytes significantly upregulated ECM deposition-related pathway “Collagen Containing Extracellular Matrix” (Fig. 4g). Additionally, in senescent tenocytes, expression of matrix metalloproteinase-2 (MMP2), a key enzyme for ECM degradation,^53–55^ was downregulated, whereas POSTN expression was upregulated (Fig. 4h and Fig. S2c). These findings suggested that senescent tenocytes exhibited impaired tissue remodeling ability and enhanced ECM deposition, thereby contributing to the inability of scar to undergo proper remodeling and promoting fibrosis progression after RCT.

To identify crucial transcription factors associated with tenocyte senescence, we performed single-cell transcriptional network analysis. Tenocyte subcluster-specific regulons were identified (Fig. S2d). We then conducted correlation analysis between regulon activity and stemness score as well as senescence score. By setting a threshold of 0.2 for correlation coefficients, we identified 14 stemness-related regulons, such as STAT2( + ), KLF10(+) and MAF(+), as well as 5 senescence-related regulons: DBP( + ), FOXO1( + ), KLF3( + ), HLF(+), and RARG(+) (Fig. 4i, j and Fig. S2e). The target network of senescence-related regulons was enriched in pathways related to cadherin binding, gliogenesis, mesenchymal transition, and protein kinase activity (Fig. 4k), indicating these pathways participated in tenocyte senescence process. Immunofluorescence further confirmed increased activation of DBP and FOXO1 in tenocytes throughout RCT progression (Fig. 4l, m). Overall, senescence-related regulons, such as DBP and FOXO1, potentially drive tenocyte senescence after RCT.

### Smooth muscle cell subclusters in RCT scar healing

We re-clustered and identified four smooth muscle cells (SMC) subclusters: myofibroblast, inflammation-related (inflam-SMC), remodeling-related (remod-SMC), and fibrosis-related (fibro-SMC) (Fig. S3a), based on characteristic gene expression (Fig. S3b) and functional enrichment (Tables S4, S6). Chemokine CCL2 and CXCL12 were highly expressed in inflam-SMC at acute stages, remodeling-related genes (MMP2 and THBS4) were highly expressed in remod-SMC, while ECM-related genes and pathways were upregulated in remod-SMC and fibro-SMC (Fig. S3d, e), indicating their distinct roles in RCT healing process. Trajectory analysis identified two developmental trajectories: myofibroblast to fibro-SMC and myofibroblast to remod-SMC (Fig. S3f), with progressively increased expression of COL1A1 and MMP2 along these trajectories (Fig. S3k). In many other diseases, SMC play an important role in scar formation.^56–58^ However, in human RCT, SMC proportions were relatively small, accounting for 6.6%, 3.3% and 0.4% at the acute, subacute and chronic stages, respectively (Fig. 2d). Consistently, the number of SMC gradually decreased as the duration of RCT progressed (Fig. S3c), suggesting that while SMC contribute to the fibrosis process after RCT, their role may be relatively less pronounced.

### Fibrosis-related macrophages involved in fibrotic scarring after RCT

Next, we focused on the profiles of immune microenvironment, including macrophages, mast cells, B cells, and T cells (Fig. S4a, b). Infiltrations of mast cells, B cells, and T cells gradually decreased, while macrophages infiltrated persistently as the duration of RCT progressed (Fig. S4c–f).

**Fig. 5:**
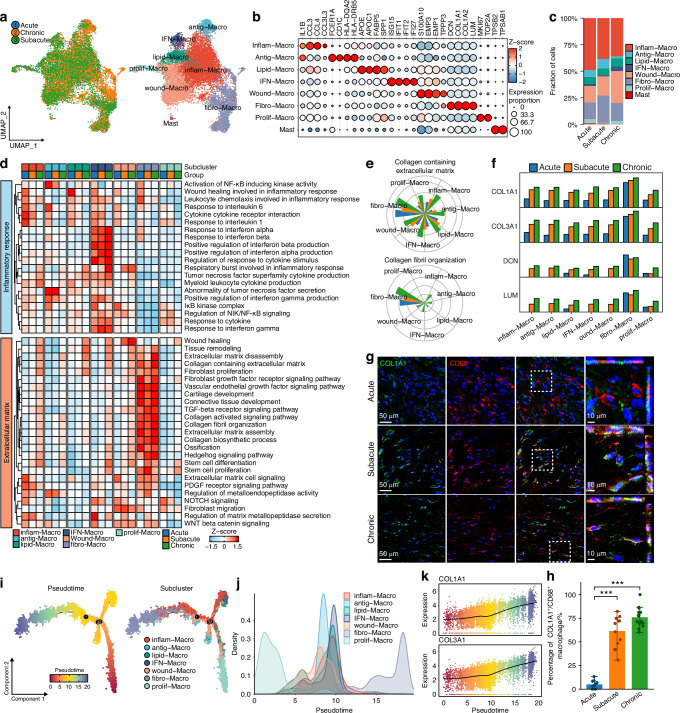
Characteristics of fibro-Macro phenotype after RCT. **a** UMAP embedding of macrophages annotated by stages and subclusters. **b** Dot heatmap showing significant upregulated genes of each subcluster. **c** Proportion of macrophage subclusters in different RCT stages. **d** Heatmap of inflammation and ECM related pathways activities in macrophage subclusters in different stages. **e** Pathway activities of “collagen containing extracellular matrix” and “collagen fibril organization” in different stages of each subcluster. **f** Bar plot showing expression of COL1A1, COL1A2, DCN and LUM in macrophage subclusters in different stages. **g** FISH and immunofluorescence co-staining showing COL1A1 expression in CD68^+^ macrophages. **h** Measurement of COL1A1^+^/CD68^+^ macrophage proportion in RCT (Acute, *n* = 10; Subacute, *n* = 13; Chronic, *n* = 15). **i** Macrophage trajectories annotated by pseudotime and subclusters. **j** Density plot showing pseudotime distribution of macrophage subclusters. **k** Expression of COL1A1 and COL3A1 along with elapsing of pseudotime in macrophages. (mean ± SD are shown as error bar, *P < 0.05, **P < 0.01, ***P < 0.001

As the persistent infiltration of macrophages, we performed re-clustering of myeloid cluster and identified seven macrophage subclusters as well as one mast cell cluster based on subcluster-specific DEGs and functional enrichment (Fig. 5a, b and Tables S4, S7). We defined the macrophage subclusters as inflammation-related (inflam-Macro), antigen presentation-related (antig-Macro), lipid metabolism-related (lipid-Macro), IFN-related (IFN-Macro), wound healing-related (wound-Macro), fibrosis-related (fibro-Macro), and proliferative (prolif-Macro) (Fig. 5a). Inflam-Macro and antig-Macro were predominantly present in the acute and subacute stages, while the proportions of fibro-Macro and wound-Macro increased in the subacute and chronic stages (Fig. 5c). Pathway activity analysis revealed that the inflammatory pathways were upregulated in inflam-Macro and antig-Macro at acute stage, while interferon-related pathways remained highly activated in IFN-Macro throughout all stages (Fig. 5d). To explore heterogeneous functions of macrophages, we performed differentially expressed genes and activated pathways analysis among different stages. Macrophages in acute stage exhibited activity of acute inflammation-related pathways, including IL-1, LPS and oxidative stress, while adaptive immune response and MHC pathways were activated in the subacute stage (Fig. S5a, b), highlighting distinct immune responses of macrophages at acute and subacute stages. Notably, macrophages in chronic stage exhibited significant activation of ECM-related pathways and upregulated expression of FRGs, indicating pro-fibrotic function (Fig. S5b, c).

Notably, we found fibro-Macro subcluster exhibited predominant upregulation of ECM-related pathways (Fig. 5d, e). Moreover, fibro-Macro highly expressed FRGs, such as COL1A1, COL3A1, DCN, and LUM (Fig. 5f and Fig. S5d). Fluorescence in situ hybridization (FISH) and immunofluorescence (IF) co-staining further validated high expression of COL1A1 in macrophages at subacute and chronic stages (Fig. 5g, h), indicating presentation of fibro-Macro phenotype, which directly expressed FRGs. Trajectory analysis revealed differentiation trajectory from prolif-Macro to inflam-Macro and wound-Macro, and ultimately culminating in fibro-Macro, indicating that fibro-Macro was differentiated from other macrophage subclusters and represented the terminal state of macrophage (Fig. 5i, j). In addition, the expressions of COL1A1 and COL3A1 gradually increased along pseudotime (Fig. 5k). These findings suggest that the fibro-Macro phenotype, as the terminal state of macrophage differentiation, directly secretes ECM components, thereby contributing to scar healing after RCT.

### SOX9 drove macrophage conversion to fibro-Macro phenotype

**Fig. 6:**
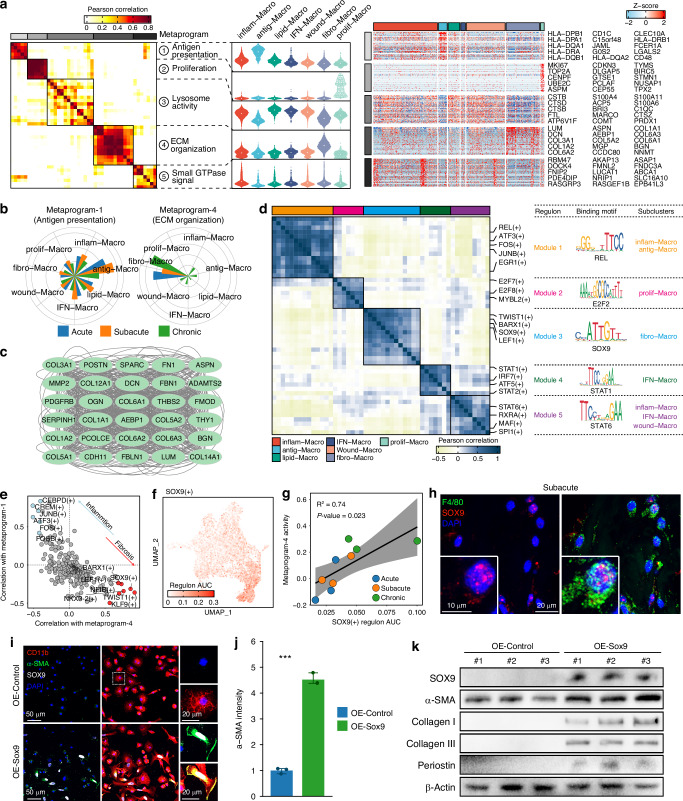
Common metaprogram and key regulators of fibro-Macro phenotype. **a** Correlation heatmap of 44 gene programs showing clustering of 5 metaprograms (left); violin plot showing metaprogram activities across macrophage subclusters (middle); expression heatmap showing co-expression patterns of top genes in each metaprogram (right). **b** Bar plot showing activities of metaprogram 1 and 4 in macrophage subclusters at different stages. **c** PPI network of hub genes of metaprogram 4. **d** Correlation heatmap of 50 regulons showing clustering of 5 co-regulated modules. **e** Correlation between each regulon and metaprogram 1 as well as 4 in macrophages. **f** UMAP embedding of macrophages showing regulon AUC of SOX9(+). **g** Dot plot showing correlation between regulon AUC of SOX9(+) and expression of metaprogram 4. **h** Immunofluorescence showing activation of SOX9 in macrophages after RCT. **i** Immunofluorescence showing expression of SOX9 and α-SMA in rat BMDMs. **j** Measurement of mean α-SMA intensity in rat BMDMs (*n* = 3). **k** Western blot analysis of α-SMA, collagen I, collagen III and periostin expression in rat BMDMs of OE-Control or OE-SOX9 groups (OE-Control, *n* = 3; OE-SOX9, *n* = 3)

To further identify gene co-expression programs of macrophage, we performed non-negative matrix factorization (NMF) and uncovered 44 co-expressed gene programs, which were then divided into 5 common meta-programs with similar gene expression patterns via hierarchical clustering (Fig. 6a). Enrichment analysis revealed that the 5 meta-programs corresponded to distinct functions of macrophages: antigen presentation, proliferation, lysosome activity, ECM organization, and small GTPase signaling (Fig. 6a and Table S8). Meta-program 1, which consisted of human leukocyte antigen (HLA) genes, represented the immune function of macrophage and showed significant upregulation in antig-Macro, while meta-program 4, associated with ECM organization, was highly expressed in fibro-Macro (Fig. 6a, b), indicating its crucial role in scar healing of RCT. We further identified the hub gene interaction network of meta-program 4, highlighting the pivot roles of COL1A1, COL3A1, COL5A2, THBS2, DCN, FN1 and other program genes in fibro-Macro (Fig. 6c).

Subsequently, we sought to identify key regulators driven the conversion to Fibro-Macro phenotype. Using SCENIC data, we clustered regulons into five co-regulated modules across macrophage phenotypes (Fig. 6d). Module 1 (including CEBPD( + ), FOS( + ), JUNB( + ), ATF3(+), etc.) was significantly activated in inflam-Macro and antig-Macro, reflecting their functions regulating macrophage immune response. In contrast, module 3 (including SOX9( + ), BARX1( + ), LEF1( + ), KLF9(+), etc.) was predominantly activated in fibro-Macro, suggesting its key role regulating fibrosis (Fig. 6d and Fig. S5e). Correlation analysis between regulons and gene co-expression metaprograms further identified inflammation-related regulons and fibrosis-related regulons, which were predominantly enriched in regulon module 1 and 3, respectively (Fig. 6e). These inflammation-related and fibrosis-related regulons potentially drove two key functions of macrophages after RCT. Among regulons in module 3, SOX9 emerged as a critical transcription factor previously reported to promote fibrosis in various diseases.^59–62^ We observed that SOX9(+) regulon activity was upregulated in fibro-Macro (Fig. 6f) and positively correlated with fibrosis-related gene metaprogram 4 (Fig. 6g). Immunofluorescence confirmed nuclear localization of SOX9 in macrophages after RCT (Fig. 6h). To validate the regulatory role of SOX9 in macrophage, we overexpressed SOX9 in rat bone marrow derived macrophages (BMDMs), confirmed by immunofluorescence and Western blot (Fig. 6i, k). SOX9-overexpressing BMDMs exhibited increased expression of α-SMA, a key marker of macrophage-myofibroblast transition (MMT),^63–65^ together with fibrosis-related ECM proteins (Collagen I, Collagen III, and Periostin) (Fig. 6i–k). Together, these findings suggest that SOX9 functions as a key regulator driving macrophage conversion to fibro-Macro phenotype and promoting ECM expression.

### Endothelial-mesenchymal transition involved in fibrotic scarring after RCT

**Fig. 7:**
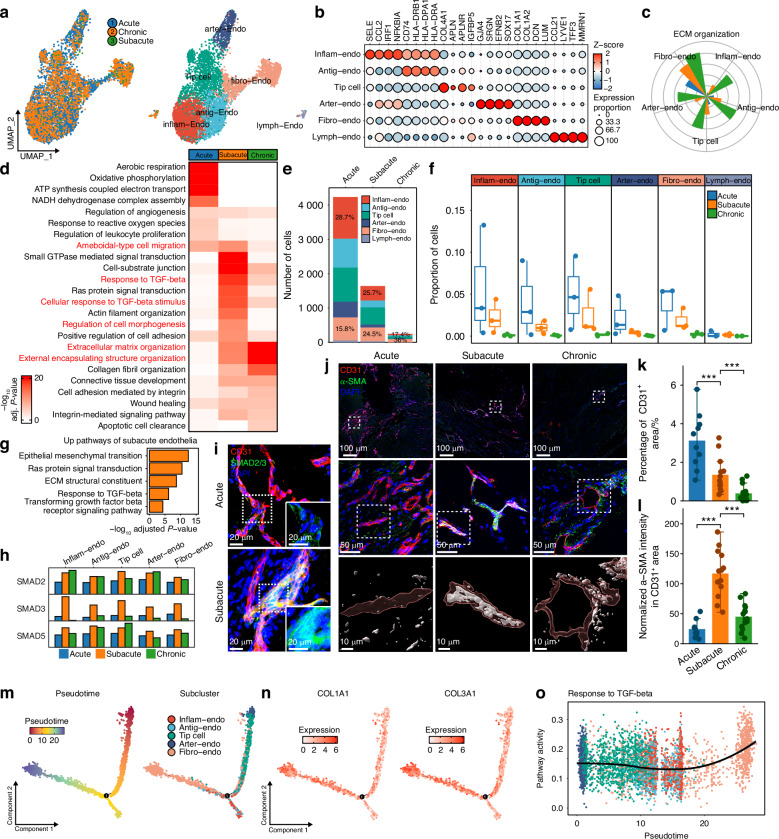
EndoMT characteristics and trajectory after RCT. **a** UMAP embedding of endothelial cells annotated by stages and subclusters. **b** Dot heatmap showing significant upregulated genes of each subcluster. **c** Pathway activity of “extracellular matrix organization” in different stages of each subcluster. **d** Heatmap showing *P*-values of enriched pathways of different stages. **e** Bar plot showing numbers of endothelial cells of different stages. **f** Box plot showing proportions of endothelial subclusters of different stages. **g** Bar plot showing upregulated pathways of subacute endothelial cells. **h** Bar plot showing expression of SMAD2, SMAD3 and SMAD5 in endothelial subclusters at different stages. **i** Immunofluorescence showing SMAD2/3 and CD31 expression in human RCT. **j** Immunofluorescence showing α-SMA and CD31 expression in human RCT. **k**, **l** Measurement of CD31^+^ area and α-SMA intensity in CD31^+^ area in human RCT (Acute, *n* = 10; Subacute, *n* = 13; Chronic, *n* = 15). **m** Endothelial trajectories annotated by pseudotime and subclusters. **n** Endothelial trajectories colored by expression of COL1A1 and COL3A1. **o** Pathway activity of “response to TGF-β” over endothelial subclusters trajectory. (mean ± SD are shown as error bar, *P < 0.05, **P < 0.01, ***P < 0.001)

We performed re-clustering and identified five vascular endothelial subclusters and a lymphatic endothelial cluster (lymph-Endo). Endothelial subclusters were annotated as inflammation-related endothelial cell (inflam-Endo), antigen presentation-related endothelial cell (antig-Endo), fibrosis-related endothelial cell (fibro-Endo), arterial endothelial cell (arter-Endo) and tip cell based on subcluster-specific DEGs and functional enrichment (Fig. 7a, b and Tables S4, S9). Inflam-Endo and antig-Endo subclusters exhibited inflammation function, whereas fibro-Endo showed high expression of FRGs and significant upregulation of ECM-related pathways (Fig. 7b, c). Functional enrichment analysis across different stages revealed that vascular endothelial cells at acute stage exhibited enhanced angiogenesis and energy metabolism. Ameboid-type cell migration and TGF-β signaling pathways were activated in the subacute stage, while ECM-related pathways were upregulated in the chronic stage (Fig. 7d).

Notably, both the number of endothelial cells and the proportions of endothelial subclusters declined markedly as RCT progressed (Fig. 7e, f), and immunofluorescence confirmed vascular regression (Fig. 7j, k). To investigate the underlying mechanism, we focused on endothelial cells during the regressing process. At the subacute stage, endothelial cells exhibited significant enrichment of mesenchymal transition and TGF-β signaling pathways (Fig. 7g), along with elevated expression of SMAD2/3/5, key mediators of TGF-β signaling (Fig. 7h). Immunofluorescence further confirmed significant upregulation of SMAD2/3 in subacute endothelial cells (Fig. 7i). Given that TGF-β signaling is a well-established driver of endothelial-mesenchymal transition (EndoMT),^66–69^ we assessed α-SMA expression, a canonical EndoMT marker, and observed its marked upregulation in subacute endothelial cells (Fig. 7j, l). These findings indicated that EndoMT contributes to vascular regression during the subacute stage of RCT. Additionally, in rat RCT model, we also observed vascular regression and significant α-SMA expression in endothelial cells across different stages (Fig. S6a–c).

Subsequently, we performed trajectory analysis and revealed a differentiation trajectory from tip cells to fibro-Endo (Fig. 7m), accompanied by progressively increased expression of COL1A1 and COL3A1 (Fig. 7n). Importantly, fibro-Endo occupied the terminal position of endothelial subclusters and exhibited high activity of TGF-β signaling (Fig. 7o). These features suggest that fibro-Endo represents a transitional state of EndoMT with a pronounced pro-fibrotic profile. By acquiring mesenchymal characteristics and highly expressing fibrosis-related ECM genes, fibro-Endo not only marks the fate of endothelial cells undergoing EndoMT but also emerges as an important cellular contributor driving fibrotic scar formation after RCT.

In addition, we explored characteristics of lymph-Endo. Differential gene expression analysis showed specific upregulation of chemokine CCL21, together with canonical lymphatic markers LYVE1 and PROX1, in lymphatic endothelial cells (Fig. S6d–f). CCL21 expression peaked in lymph-Endo at the acute stage and gradually declined thereafter (Fig. S6g), which was further validated by FISH and immunofluorescence co-staining (Fig. S6h, l). Moreover, immunofluorescence showed the highest percentage of Ki67^+^ lymph-Endo, consistent with active proliferation, and the highest density of lymphatic vessels at the subacute stage (Fig. S6f–h). These findings suggested that lymph-Endo may promote inflammatory process via CCL21 secretion, and their regression occurred later than that of vascular endothelial cells, highlighting distinct temporal dynamics of lymphatic versus vascular remodeling.

### OPN and TGF-β signaling promoted fibrotic scarring after RCT

**Fig. 8:**
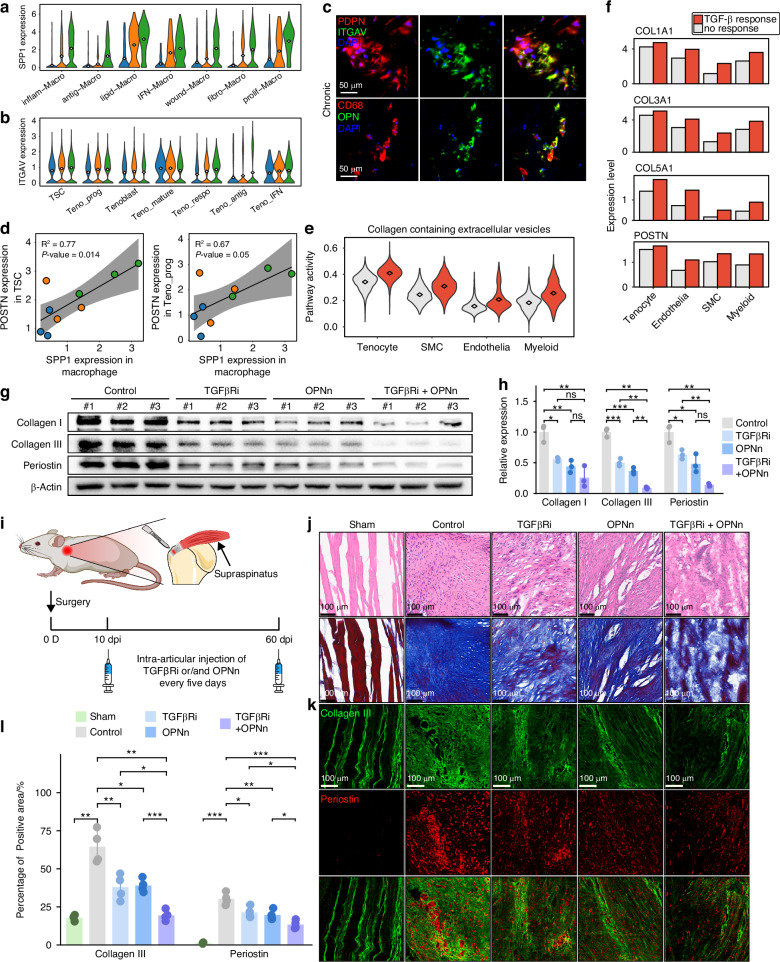
OPN and TGF-β signaling induced fibrotic scarring after human RCT. **a** Violin plot showing expression of SPP1 in macrophage subclusters. **b** Violin plot showing expression of ITGAV in tenocyte subclusters. **c** Immunofluorescence of RCT sample at the chronic stage showing expression of OPN in macrophages and ITGAV in tendon stem cells. **d** Correlation analysis between SPP1 expression in macrophages and POSTN expression in TSC as well as Teno_prog. **e** Violin plot showing activity of pathway “collagen containing extracellular matrix” in TGF-β response and no-response cells. **f** Bar plot showing FRGs expression in TGF-β response and no-response cells. **g**, **h** Western blot analysis of collagen I, collagen III and periostin expression in administration of OPNn and/or TGFβRi groups of primary rat tenocytes treated with OPN/TGF-β (*n* = 3). **i** Diagram of rat supraspinatus tendon rupture model and intra-articular injection of OPNn and TGFβRi. **j** Histological imaging of H&E and Masson’s staining in different treated groups of rat RCT model (*n* = 4). **k** Immunofluorescence labeling Collagen III and periostin expression in different treated groups of rat RCT model. **l** Measurement of Collagen III^+^ and periostin^+^ area in different treated groups of rat RCT model (*n* = 4). (mean ± SD are shown as error bar, *P < 0.05, **P < 0.01, ***P < 0.001)

To explore pro-fibrotic signaling in the RCT microenvironment, we first conducted intercellular communication analysis across subclusters, which revealed extensive crosstalk among different subclusters (Fig. S7a). Given that macrophage and tenocyte were the predominant cell types in RCT, we focused on macrophage-tenocyte interactions. Among the detected signals, interactions between SPP1 (encoded OPN protein) and its receptors ITGAV, CD44, ITGA5, ITGB1 and ITGB5 were the most prominent (Fig. S7b). SPP1 was predominantly expressed in macrophage subclusters at chronic stage (Fig. 8a), while its receptors were broadly expressed across tenocyte subclusters (Fig. 8b and Fig. S7c). Immunofluorescence confirmed OPN expression in macrophage and ITGAV expression in TSC at the chronic stage of RCT (Fig. 8c). Furthermore, the expression pattern of SPP1 in macrophages closely positively correlated with that of POSTN in TSC as well as Teno_prog (Figs. 8d, 3k), indicating that OPN may promote ECM secretion by tenocyte subclusters.

On the other hand, we summarized six canonical signaling pathways known to regulate tissue healing and fibrosis in various diseases, including TGF-β, bone morphogenetic protein (BMP), fibroblast growth factor (FGF), platelet-derived growth factor (PDGF), epidermal growth factor (EGF) and vascular endothelial growth factor (VEGF) signaling. We first evaluated the response activity of subclusters to these pathways. The main responding cells included tenocyte, SMC and endothelial subclusters (Fig. S7d). Specifically, TSC, Teno_prog, Teno_respo, remod-SMC, fibro-SMC, fibro-Macro and fibro-Endo exhibited significant response proportions to these signaling (Fig. S7d and Fig. S8a), reflecting extensive pro-fibrotic interactions within the RCT microenvironment. We next examined the source of key regulatory ligands for these signaling. Tenocytes were the predominant source of most of these factors, such as family members of TGFB, BMP, PDGFA and VEGF, as well as connective tissue growth factor (CTGF), a potent pro-fibrotic mediator that activates TGF-β and BMP signaling,^70^ while macrophages also contributed substantially to TGFB, PDGF, and VEGF production. (Fig. S7d). Furthermore, cells exhibiting high responsiveness to TGF-β signaling showed significantly increased expression of fibrosis-related genes (FRGs) and elevated ECM-related pathway activity compared with low-responsiveness cells (Fig. 8e, f). Response to BMP and VEGF signaling also enhanced FRG expression, whereas FGF, PDGF, and EGF signaling appeared to exert relatively modest effects on FRG expression in most subclusters other than SMC (Fig. S8b).

To further identify roles of OPN and TGF-β signaling, we treated primary tenocytes with recombinant OPN and TGF-β1 proteins in vitro. Both OPN and TGF-β1 treatment significantly upregulated the expression of collagen I, collagen III and periostin in tenocytes. Moreover, combined treatment with OPN and TGF-β1 induced a higher increase in ECM protein expression (Fig. S7e, f). These findings suggest that OPN and TGF-β signaling act synergistically to promote ECM production in tenocytes, particularly during the subacute and chronic stages, thereby driving sustained ECM deposition and scar formation after RCT.

To investigate the profibrotic roles of OPN and TGF-β signaling in vivo, we applied OPN neutralizing antibody (OPNn) to block OPN activity, and SB-431542, a TGF-β receptor kinase inhibitor (TGFβRi), to inhibit TGF-β receptor signaling. First, primary rat tenocytes were pre-treated with OPNn, TGFβRi, or their combination for 1 h, followed by OPN and TGF-β1 administration. Both OPNn and TGFβRi significantly reduced the expression of collagen I, collagen III, and periostin, with combined treatment producing the most pronounced reduction in ECM protein levels (Fig. 8g, h), confirming the effectiveness of OPNn and TGFβi in vitro. Subsequently, we performed intra-articular injection of OPNn or/and TGFβRi for rat RCT model. Considering that upregulation of OPN and TGF-β signaling begins at the subacute stage, injection was initiated at 10 days post-injury (Fig. 8i). H&E and Masson’s staining revealed that scar tissues in OPNn- or TGFβRi-treated groups were looser comparing with the dense scar in untreated group (Fig. 8j). Immunofluorescence further illustrated that Collagen III and periostin expression was substantially reduced in OPNn- or TGFβRi-treated groups, with the lowest expression observed in the combined treatment group (Fig. 8k, l). These findings confirmed the pro-fibrotic roles of OPN and TGF-β signaling after RCT, and highlighted the potential of targeting these pathways as anti-fibrotic strategies.

## Discussion

Although previous studies have revealed various pathways and mediators involved in scar healing after rotator cuff tear (RCT),^71–73^ the overall mechanisms of injury microenvironment after human RCT contributes to fibrotic repair remain unclear. This study profiled scar characteristics and constructed cellular and molecular dynamics of human RCT. By conducting scRNA-seq, we identified several previously unrecognized cellular phenotypes and states involved in scar formation. These findings provide a landscape for comprehensive understanding of the pivotal mechanisms underlying scar healing of human RCT.

Our results reveal the dynamic features of fibrotic scarring across different stages of RCT. First, we demonstrate that human RCT repair results in persistent scar formation characterized by disrupted tendon architecture, disorganized collagen alignment, imbalanced type I/III collagen ratio, and reduced collagen fibril diameters. Second, we decode the dynamical characteristics and heterogeneities of various cell types involved in scar formation of human RCT based on scRNA-seq analysis and experimental validation.

Our data suggest that tendon stem cells (TSCs) and tendon progenitor cells (Teno_prog) are the main sources of ECM protein secretion. After human RCT, TSCs and Teno_prog proliferate actively. However, the majority of TSCs and Teno_prog does not differentiate into mature tenocytes; instead, they exist at the subacute and chronic stages of RCT and secrete extracellular matrix protein in large amounts continuously, leading to persistent ECM deposition. Notably, we observed significant expression of POSTN in tenocytes and significant deposition of periostin at subacute and chronic stages. Given that periostin is an important pro-fibrotic ECM component,^74^ it may also contribute to scar formation of human RCT, which warrant further exploration.

Studies have shown that cellular senescence is a critical driver of fibrosis progression.^47,75^ Our data observed progressive senescence of tenocytes after human RCT, which was accompanied by impaired abilities of tissue remodeling and ECM degrading. We further identified significant activation of transcription factors DBP, FOXO1, KLF3, HLF, and RARG in senescent tenocyte. Further studies are needed to determine whether these regulators driven tenocyte senescence following human RCT.

Inflammation cascade is another driver contributing to fibrosis progression.^76,77^ Immune infiltration brings in situ inflammation, debris clearance and death of functional cells, which activate pro-fibrotic signaling.^10^ We discover significant infiltration of immune cells at the acute stage of human RCT, including macrophages, T cells, B cells, and mast cells. These immune components could be chemoattracted to the injured tendon through newly formed blood and lymphatic vessels potentially mediated by chemokines such as CCL21 secreted from lymphatic endothelial cells. Distinct pro-inflammatory phenotypes, such as Teno_antig, Teno_IFN, inflam-SMC, inflam-Macro, and inflam-Endo, may also promote chemotaxis and infiltration of immune cells.

As expected, the majority of immune cells subsides as the duration of RCT progressed. However, at the subacute and chronic stages, macrophages infiltrate persistently, exhibiting repressed inflammatory activity and a pro-fibrotic pattern with high expression of ECM-related genes and pathways, which we termed as fibro-Macro phenotype. Phenotype conversion from inflammatory to pro-fibrotic macrophages could result in excessive ECM deposition, thereby accelerating scar formation. Fibro-Macro observed in human RCT shares similarities with scar-associated macrophages found in other fibrotic diseases^17,18^ but also exhibits distinct differences, representing a newly identified macrophage phenotype. We further identified SOX9 as the key transcription factors of fibro-Macro, and overexpression of SOX9 gene in macrophages could induce fibro-Macro phenotype in vitro, indicating SOX9 drives the phenotype conversion to fibro-Macro. Besides, at the subacute and chronic stages, macrophages secreted OPN in large amounts, which could have effect on tenocytes via ligand-receptor interaction and promote ECM secretion of tenocytes. These results indicate that macrophages are heterogeneous and may promote scar formation after human RCT through direct or indirect mechanisms.

Normal tendon is avascular tissue.^78,79^ However, we observed that RCT boosts angiogenesis during acute stage. The newly formed vasculature is immature and leaky, delivering blood supply alongside excessive inflammation components. Our findings suggest that endothelial cells undergo endothelial-to-mesenchymal transition (EndoMT), mediated by TGF-β signaling, during the subacute stage, which may explain the regression of vasculature. We further identified an intermediate phenotype of EndoMT process--fibro-Endo, characterized by high expression of ECM-related genes and pathways and may participate in scar repairing of RCT. Studies suggest that limited angiogenesis and timely vascular regression are believed to mitigate scar formation.^79^ However, during the natural healing process of human RCT, vascular regression does not coincide with reduced scar formation. This may be attributed to the delayed timing of vascular regression or the promotion of scar formation by fibro-Endo phenotype. In summary, the relationship between vascular regression and scar formation in RCT is complicated.

Scar-free healing of human RCT relies on three critical factors: limited inflammation, moderate ECM deposition, and sufficient tissue remodeling capacity. However, during the natural healing process of adult RCT, these prerequisites are disrupted due to various challenges. First, persistent infiltration of macrophages and other pro-inflammatory phenotypes sustains a smoldering inflammation microenvironment in the injury site of RCT, which cause tissue damage and activation of pro-fibrotic signaling. Second, various cells converted into ECM-secreting phenotypes after RCT, such as TSC, Teno_prog, fibro-SMC, fibro-Macro, and fibro-Endo, which respond to multiple pro-fibrotic signaling, leading to persistent and robust ECM deposition. Third, along with the disease progression, remodeling capacity were impaired by cellular senescence, causing the permanent and chaotic deposition of ECM components which could not be replaced.

In conclusion, our study provides comprehensive insights into scar formation of natural healing process of human RCT and several targets for reversing scar. However, several limitations should be noted in this study. First, the difficulty in obtaining high-quality clinical tendon stump samples restricted the sequencing sample size. Second, semitendinosus tendon, rather than healthy rotator cuff tissue, was used as the control. Third, although standard quality control and normalization procedures were applied, potential batch effects in single-cell sequencing cannot be excluded. Finally, the study was based solely on transcriptomic data, which may not fully reflect protein-level changes. Further investigations of the origin, characteristics and conversion of these cell phenotypes should provide more insights into the fundamental mechanisms underlying scar formation in human rotator cuff tear.

## Materials And Methods

### Clinical information and samples collection

This study was approved by the Clinical Medical Ethics Committee of Xiangya Hospital, Central South University. After applying the inclusion and exclusion criteria and obtaining patients’ written informed consent, 38 patients (10, 13, and 15 cases of acute, subacute, and chronic RCT) and 2 patients with anterior cruciate ligament (ACL) tear, who were diagnosed with RCT or ACL tear at Xiangya Hospital, Central South University, were enrolled. Standard arthroscopic techniques were used to obtain supraspinatus tendon tear stump (approximately 0.5 cm^3^) from the 38 RCT patients undergoing surgery, and semitendinosus tendon tissues were obtained as healthy controls from 2 patients undergoing ACL reconstruction. scRNA-seq was performed on samples from 9 RCT patients with different disease stages and matched clinical characteristics. Clinical and demographic information of all patients, and detailed inclusion/exclusion criteria and definitions of different RCT stages are summarized and provided in Table S1. The definition of three different stages of rotator cuff tear was categorized as follows: Acute phase: disease duration ≤ 6 weeks; Subacute phase: > 6 weeks and < 6 months; Chronic phase: ≥ 6 months.

### Single-cell RNA-seq data analysis

Raw Fastq sequencing files were processed and aligned to reference genome GRCh38 by 10x Genomics CellRanger software (version 6.1.2) with default parameters. The gene expression matrix for each sample was integrated and converted into Seurat objects via the R package Seurat (version 4.2.1).^80^ Cells with >200 & <6 000 detected genes and <20% of mitochondrial genes expression proportions were retained. To eliminate potential doublets, R package Doubletfinder (version 2.0.3) was applied to each sample individually. We set pN = 0.25 and optimized pK for each sample using the parameter sweep function. The expected number of doublets (nExp) was calculated based on the multiplet rate estimated from the 10x Genomics guideline (~0.8% per 1 000 recovered cells). R package harmony (version 0.1.0) was used to remove batch effect across different patient samples with the top 30 principal components (PCs), theta = 2, lambda = 1, and max_iter = 20. After quality control, a total of 87 730 high quality cells were then normalized and scaled, the top 5 000 variable genes were identified, and Principal component analysis (PCA) was performed. Uniform Manifold Approximation and Projection (UMAP) and cell clustering were performed based on top 50 PCA components. Clusters were annotated to major cell types based on the combination of (i) machine algorithm SingleR (version 2.0.0) with the Human Primary Cell Atlas reference and (ii) manual curation by expression of canonical marker genes for major cell type from published single-cell studies (Fig. 2c, Table S2).^81–83^ Canonical Correlation Analysis (CCA) algorithm was then used for further correction of each cell type with top 3 000 variable genes and top 30 PCs. Detail descriptions for methods of subclusters annotation, fibrosis-related genes collection, RNA velocity, cell trajectory, single cell senescence evaluation, SCENIC, gene co-expression network and intercellular communication analysis were displayed in supplementary materials.

### Tenocyte stimulation and inhibition assays

For TGF-β1 and OPN stimulation assay, primary rat tenocytes were assigned to four groups: Control; TGF-β1 group, treated with recombinant TGF-β1 protein (MedChemExpress, HY-P7117, 10 ng/mL); OPN group, treated with recombinant OPN protein (MedChemExpress, HY-P79222, 1 μg/mL); TGF-β1 + OPN group, treated with both recombinant TGF-β1 (10 ng/mL) and OPN (1 μg/mL) for 48 h. For TGFβRi and OPNn inhibition assay, cells were assigned to four groups: Control; TGF-β receptor inhibitor (TGFβRi), pre-treated with SB-431542 (MedChemExpress, HY-10431,10 μmol/L) for 1 h; OPN neutralizing antibody (OPNn), pre-treated with OPNn (Bioxcell, BE0382, 10 μg/mL) for 1 h; TGFβRi + OPNn, pre-treated with both SB-431542 (10 μmol/L) and OPNn (20 μg/mL) for 1 h. Following pre-treatment, all groups were stimulated with TGF-β1 (10 ng/mL) and OPN (1 μg/mL) simultaneously while maintaining the inhibitors in the medium, and incubated for 48 h. ECM-related genes expression was then measured by western blotting.

### Construction of rat rotator cuff injury model

All animal procedures were approved by the Animal Ethics Committee of Central South University. Male Sprague–Dawley rats (approximately 300 g) were used to establish a rotator cuff injury (RCT) model. Surgeries were performed by a clinician with extensive anatomical knowledge and proficient surgical skills to minimize variability. Briefly, rats were anesthetized with continuous inhalation of isoflurane and positioned to fully expose the left shoulder. A longitudinal incision was made, and the muscles and fascia were carefully separated using blunt dissection. The supraspinatus tendon was identified, lifted with a needle, and transected from the humeral head without suturing to mimic a clinical supraspinatus tear. Muscles and skin were sutured layer-by-layer to complete the procedure. Sham-operated controls underwent identical procedures without tendon transection. For intervention experiments, rats were randomly assigned to control, TGFβ receptor inhibitor (TGFβRi), OPN neutralizing antibody (OPNn), and combination groups. From 10 days post-injury, corresponding to the subacute stage of tendon healing, rats received 20 μL intra-articular injections every 5 days until 60 days post-injury: PBS (control), SB-431542 (MedChemExpress, HY-10431, 10 μg/shoulder), OPNn (Bioxcell, BE0382, 20 μg/shoulder), or SB-431542 + OPNn. Rats were used then euthanized for further analysis.

### Statistical analysis

Statistical analyses were conducted using R software (version 4.1.3). For comparisons of continuous variables, non-parametric tests including the Wilcoxon rank-sum test for two groups and the Kruskal–Wallis test for multiple groups, and parametric tests including Student’s *t* test for two groups and ANOVA test for multiple groups, were applied as appropriate. Pearson’s correlation analysis was used to assess the relationship between continuous variables. For statistical tests involving multiple comparisons, *P*-values were adjusted using the Benjamini–Hochberg (BH) method to control the false discovery rate (FDR). Specifically, FDR correction was applied in (i) differential gene expression identification using Seurat FindAllMarkers, (ii) GO/KEGG functional enrichment analyses using clusterProfiler, (iii) intercellular ligand-receptor interaction significance using CellChat. All “adjusted *P*-values” present in Result section refer to FDR-adjusted *P*-values. Results with *P* value < 0.05 or FDR-adjusted *P* < 0.05 were considered statistically significant unless otherwise specified.

## Supplementary information

Supplementary Materials

Supplementary Table S1

Supplementary Table S2

Supplementary Table S3

Supplementary Table S4

Supplementary Table S5

Supplementary Table S6

Supplementary Table S7

Supplementary Table S8

Supplementary Table S9

Supplementary Table S10

Supplementary Figure S1

Supplementary Figure S2

Supplementary Figure S3

Supplementary Figure S4

Supplementary Figure S5

Supplementary Figure S6

Supplementary Figure S7

Supplementary Figure S8

Original WB images

## Data Availability

All the data support the figures and the other findings are available upon reasonable request to the corresponding author.
