# Supplementary material for: Single cell atlas decodes the molecular dynamics of scar repair after human rotator cuff tear
Source: Bone Res. 2026 Feb 5;14:17. doi: 10.1038/s41413-025-00501-5 (PMC12877062; doi:10.1038/s41413-025-00501-5)
Supplement: Supplementary file 1 — Supplementary Materials [file 41413_2025_501_MOESM1_ESM.docx]

**Supplementary methods**

**Magnetic resonance imaging (MRI)**

MRI scans were performed using a GE Discovery MR750w 3.0T scanner equipped with an 8-channel shoulder coil. The imaging protocol included oblique coronal fat-suppressed proton density-weighted (PD-FS), oblique sagittal T1-weighted, and oblique sagittal PD-FS sequences. Typical parameters were slice thickness 3.0–4.0 mm with interslice gap 0.5–1.0 mm, field of view (FOV) 17–20 cm, in-plane resolution 0.5–0.8 mm, TR 2100–3100 ms, TE 35–42 ms, and echo train length 10–12. MRI was used for diagnosis of RCT and identification of tear type. Tear type was classified as full-thickness tear, defined as complete discontinuity of the tendon from the articular to the bursal side with a high-intensity signal traversing the entire tendon thickness on T2WI/PD-FS sequences, or partial-thickness tear, involving only a portion of the tendon thickness.

**Tissue processing**

Fresh tendon stump tissues were washed 3 times with 1ⅹPBS, cut into small pieces, and digested with diluted 2 mg/ml type I collagenase (Sigma-Aldrich, C0130) and 1 mg/ml type II collagenase (Sigma-Aldrich, C2-28) in low-glucose DMEM (Gibco, 11885084) at 37℃ for 60 minutes. The digestion was terminated with an equal volume of complete medium containing fetal bovine serum. Cells were filtrated through a 70 µm cell strainer and centrifuged at 500g for 5 minutes. Red blood cells were lysed for 10 minutes using Lysing Buffer (BD, 555899), washed with PBS for 2 times, and then centrifuged 500g for 5 minutes. Cell viability was calculated using trypan blue staining. Only samples with >85% viability were selected for following sequencing. The qualified cells were resuspended at a concentration of 700-1200 cells/µl for processing on the 10x Genomics Chromium™ system.

**Histological staining**

Tendon samples were fixed and dehydrated, then 10 µm frozen sections were prepared using OCT embedding. These sections are used for H&E, Masson, safranin O-fast green, picrosirius red staining. For H&E staining, hematoxylin stained the nuclei and eosin stained the cytoplasm, for the observation of structure of tendon tissue. Masson staining, using fuchsin and aniline blue, highlighted collagen fiber distribution. For safranin O-fast green staining, safranin O and fast green dyes were used to stain cartilage and tendon tissue, respectively. Picrosirius Red staining visualized collagen networks and components. Polarized light microscopy captured images for picrosirius red-stained sections, while other images were obtained via optical microscopy.

**Transmission electron microscopy (TEM)**

The tissue specimens were fixed using standard protocols to assess collagen fiber diameter by TEM. The specimens were prefixed with 2% glutaraldehyde for 24 hours, washed twice with phosphate-buffered saline (PBS), and then fixed with 1% osmium tetroxide for 2 hours. After washing twice in PBS, the specimen was dehydrated in an ethanol gradient and dried to critical point. The specimens were then cut into ultra-thin sections with a thickness of 60-80nm, and the ultrastructure of collagen fibers was observed by TEM after heavy metal staining.

**scRNA-seq library preparation and next-generation sequencing**. An appropriate volume of cell suspension was calculated to contain 8000 ~10,000 cells for each sample. Single-cell capturing and library construction were performed using the 10x Chromium Single Cell Reagent Kit v3.1(PN-1000128, 10x Genomics) according to the manufacturer’s instructions. Briefly, the cell suspension, barcoded gel beads and partitioning oil were loaded onto the 10x Genomics Chromium Chip K to generate single-cell Gel Beads-in-Emulsion (GEMs). Captured cells were lysed and the transcripts were barcoded through reverse transcription inside individual GEMs. Then cDNA along with cell barcodes were PCR-amplified. Library construction and deep sequencing were performed by Genergy Biotechnology Co. Ltd. (Shanghai, China) using Illumina nova6000 following the manufacturer’s instructions (Illumina).

**Subclusters annotation**

For functional subcluster annotation, each major cell type was re-clustered into finer subpopulations using the Seurat package (version 4.2.1). PCA was first applied, and the number of significant PCs was determined by the elbow plot and JackStraw test. Clustering was then performed using the FindClusters function with the Louvain algorithm. The optimal resolution parameter was empirically chosen within the range of 0.2–1.2 by evaluating cluster stability, silhouette scores, and biological interpretability, ensuring that each subcluster contained a sufficient number of cells (>50) while avoiding over-fragmentation. Differentially expressed genes (DEGs) for each subcluster were identified using the Seurat FindAllMarkers function (Wilcoxon rank-sum test, adjusted p < 0.05, |log2 fold change| > 0.25, min.pct = 0.1). Functional enrichment analysis was performed on subcluster-specific DEGs using Gene Ontology (GO) analysis implemented in the clusterProfiler package (v4.2.2) together with org.Hs.eg.db (v3.16.0). Based on subcluster-specific DEGs and enriched biological processes, each cell type was annotated with distinct functional subclusters. The subcluster-specific DEGs and GO enrichment results of subclusters were summarized in supplementary tables.

**Construction of fibrosis-related genes**

A curated panel of eight fibrosis-related genes (COL1A1, COL1A2, COL3A1, COL5A2, FN1, DCN, LUM, POSTN) was used to assess fibrotic signatures. These genes were selected based on their well-established roles in extracellular matrix (ECM) production, collagen fibril organization, and tissue scarring, as supported by prior fibrosis studies (Table S3) and pathway databases (GO term “extracellular matrix organization” and “Collagen fibril organization”).

**RNA velocity analysis**

The Seurat object was converted to an AnnData object and loaded using python software Scanpy (version 1.9.6). Velocyto (version 0.17.17) was run to process all bam files, with genes.gtf file from the GRCh38 reference of CellRanger and the GRCh38 repeatmasker.gtf file downloaded from UCSC Genome Browser (genome.ucsc.edu) as input gtf files, quantifying spliced and unspliced mRNA counts. The output loom files were combined and loaded into python software scVelo (version 0.2.4) and merged with AnnData object to calculate the RNA velocity vector and visualize the RNA velocity trajectory. For RNA velocity estimation, scvelo.tl.velocity function was applied with "stochastic" mode.

**Cell trajectory analysis and CytoTRACE2**

R package Monocle (version 2.28.0) was used to construct developmental trajectories of cell populations. A CellDataSet (CDS) object was generated from the raw count matrix of top 3000 variable genes of Seurat object with the parameters expressionFamily = negbinomial.size and lowerDetectionLimit = 0.5. SizeFactors and dispersions were estimated using default settings. Dimensionality reduction for trajectory inference was performed with DDRTree algorithm, and cells were subsequently ordered in pseudotime without predefining a root state. R package CytoTRACE2 (version 1.0.0) was used to evaluate the differentiation potential of cells based on the raw count matrix of Seurat object with default parameters. Pseudotime and CytoTRACE2 scores were then presented on the DDRTree trajectory to infer both pseudotime progression and differentiation potency.

**Single cell senescence evaluation**

We used python package SenCID (version 1.0.0) to calculated senescence scores for tenocytes at single cell level. The normalized gene–cell expression matrix was used as input, and senescence identity (SID) groups as well as corresponding SID scores were computed following the developer’s recommended workflow. Among the SID groups, SID_5 group was selected for tenocytes because it showed the highest compatibility with known senescence-associated gene expression signatures. The resulting SID scores were then projected onto the DDRTree trajectory to visualize the distribution of senescence potential along pseudotime. Default parameters were applied unless otherwise specified.

**Single cell regulatory network identification**

To construct single cell transcriptional factors (TFs) regulatory networks, python package pySCENIC (version 0.12.1) was applied. Three requisite data, including list of human TFs (Hg38), whole genome ranking database of human (hg38_10kbp_up_10kbp_down_full_tx_v10_clust.genes_vs_motifs.rankings.feather) and motif annotation to transcription factors (motifs-v10nr_clust-nr.hgnc-m0.001-o0.0.tbl), were downloaded from cistarget database (<https://resources.aertslab.org/cistarget/>). First, genes expressed in fewer than 3% of cells were filtered out, and the scRNA-seq expression matrix was normalized to infer gene regulatory networks using GRNBoost2 algorithm with default parameters. Second, indirect targets for each TFs were excluded based on motif annotation via cisTarget algorithm. Third, the regulon activities were quantified for each cell via AUCell algorithm. A regulon was defined as a gene regulatory network that a TF regulates a series of targets genes directly. To identify stemness/senescence-related regulons in tenocytes, Pearson correlation analysis was performed among regulons and stemness/senescence scores. Regulons with correlation coefficients > 0.2 with the stemness score and < -0.2 with the senescence score were defined as “stemness-related regulons,” whereas regulons with a correlation > 0.2 with the senescence score and < -0.2 with the stemness score were defined as “senescence-related regulons”. To identify co-regulated modules in macrophages, Pearson correlation analysis was performed among all regulons, and regulons with correlation coefficients > 0.3 with at least two other regulons were retained. Those retained regulons were then subjected to Pearson correlation analysis and hierarchical clustering again, and regulons which clustered into one cluster were defined as a co-regulated module. The binding motifs of TFs were downloaded from JASPAR database (<https://jaspar.elixir.no/>).

**Single cell gene co-expression network identification**

To identify gene co-expression programs, non-negative matrix factorization (NMF) algorithm was used via python package cNMF (version 1.4.1). Genes expressed in fewer than 3% of cells were filtered out and NMF algorithm was performed to raw count matrix to identify gene programs for each sample independently. We retained programs with a standard deviation of cell scores greater than 0.8. A higher standard deviation indicates that the program contributes to meaningful heterogeneity across cells rather than representing uniformly expressed background signals. Pearson correlation analysis and hierarchical clustering were then performed across retained programs. Programs which clustered into one cluster were grouped as one meta-program. Six meta-programs were finally identified for macrophages. Top 100 hub genes of each meta-program were identified and enrichment analysis was performed for each meta-program to define its function (Table S8).

**Intercellular communication analysis**

Intercellular communication analysis between subclusters was performed using R package CellChat (version 1.6.1). The normalized expression matrix and corresponding subcluster annotations were used to create a CellChat object, and genes expressed in fewer than 3% of cells were filtered out. Ligand-receptor interactions were inferred based on the CellChatDB.human database (version 2020). Communication probabilities were calculated using computeCommunProb function with raw.use = TRUE and default settings, and filterCommunication function was applied to exclude communication involving clusters with very few cells (min.cells = 10). computeCommunProbPathway and aggregateNet function were applied to aggregate signaling at the pathway level. Interactions between macrophage and tenocyte subclusters, as well as between pro-inflammatory and immune subclusters, were analyzed.

**Primary rat bone marrow-derived macrophages (BMDMs) isolation and cultivation and Sox9 overexpression**

Male Sprague–Dawley rats (approximately 300g) were euthanized by excessive isoflurane inhalation. Femurs and tibias were aseptically dissected, and bone marrow cells were flushed out with ice-cold PBS using a 50ml syringe. The collected cells were filtered through a 70 μm cell strainer, centrifuged at 300 × g for 5 min, and resuspended in RPMI 1640 medium supplemented with 10% FBS, 1% penicillin/streptomycin, and 20 ng/mL recombinant M-CSF (Novoprotein, C470). Cells were plated in non-coated culture dishes and incubated at 37°C with 5% CO₂. After 7 days, adherent BMDMs were harvested for downstream experiments. For Sox9 overexpression, BMDMs were transduced with pcSLenti-CMV-Sox9-3xFLAG-PGK-Puro-WPRE3 lentivirus at a multiplicity of infection (MOI) of 40 in the presence of 8 μg/mL polybrene. After 24 h, the medium was replaced with fresh RPMI 1640 containing 10% FBS. At 72h post-transduction, puromycin (2 μg/mL) was added for selection for 3 days. Overexpression efficiency was confirmed by western blotting.

**Primary rat tenocytes isolation and cultivation**

Male Sprague–Dawley rats (approximately 300g) were euthanized by excessive isoflurane inhalation. Supraspinatus tendon was immediately harvested and washed with PBS and cut into about 1 mm^3^ in EP tubes, then washed three times with PBS. A digestion mixture (containing 2 mg/ml type I collagenase (Sigma-Aldrich, C0130), 1 mg/ml type II collagenase (Sigma-Aldrich, C2-28), 1 mg/ml dispase II (Roche, 04942078001), and 20 ng/ml DNase I (Roche, 10104159001)) was added to the tissue. The mixture was placed in a 15 mL centrifuge tube with the lid unscrewed and digested at 37°C with shaking at 180 rpm for 1 hour. Digestion was terminated by adding an equal volume of complete medium and filtration through a 70 μm cell filter. Filtrate was collected and centrifuged at 300 g for 5 minutes at 4°C. After discarding the supernatant, red blood cells were lysed for 5 minutes, and centrifuged again after termination and discarding the supernatant. The cell pellet was resuspended in complete medium and seeded into a 6-well plate. The cells were cultured at 37°C with 5% CO₂ and the medium was changed every two days.

**Immunofluorescence staining**

Immunofluorescence (IF) was performed on 10 μm frozen sections of tendon stump specimens. After rewarming and fixation, sections were washed with PBS for 3 times, blocked with 3% bovine serum album for 30 minutes, and permeated with 0.1% Triton-X for 30 minutes, at room temperature. Subsequently, sections were stained with primary antibodies for PDPN (Proteintech, 11629, 1:200), CD248 (Proteintech, 60170, 1:100), Periostin (For human specimens: Proteintech, 19899, 1:200; for rat specimens: Proteintech, 66491, 1:200), DBP (Proteintech, 12662, 1:200), FOXO1 (Cell Signaling Technology, 2880, 1:100), CD68 (GeneTex, FA-11, 1:200), F4/80 (abcam, ab16911, 1:400), SOX9 (abcam, ab185966, 1:400), CD31 (R&D Systems, AF3628, 1:400), α-SMA (abcam, ab32575, 1:400), SMAD2/3 (Cell Signaling Technology, 8685, 1:400), ITGAV (abcam, ab179475, 1:200), OPN (abcam, ab11503, 1:400), LYVE-1 (R&D Systems, AF2089, 1:400), Collagen III (Proteintech, 22734, 1:200) and Ki67 (abcam, ab15580, 1:400) overnight at 4℃, followed by conjugated secondary antibodies incubation for 2 hours at room temperature. Fluoroshield with DAPI (GeneTex, GTX30920) was then used for nuclear staining and section mounting. Fluorescent imaging was captured using ApoTome.2 microscope (Zeiss).

**Multiplexed immunofluorescence staining**

Multiplex staining was performed using Color Multiple Fluorescence Kit (AiFang Biological) according to the manufacturer’s instructions. In brief, after antigen retrieval, the slides were sequentially incubated with primary antibodies, followed by HRP polymer-conjugated secondary antibody incubation and tyramide signal amplification. Each primary antibody was stained stepwise using the same protocol. Nuclei were counterstained with Fluoroshield with DAPI (GeneTex, GTX30920). Multispectral images were acquired with Akoya Biosciences PhenoImager™ HT and analyzed using the inForm™ image analysis software (PerkinElmer).

**Fluorescent in situ hybridization (FISH)**

RNA in situ hybridization was performed on frozen tissue sections using CY3 RNASweAMI™In situ hybridization fluorescence detection kit (Servicebio) according to the manufacturer's protocol. Briefly, sections were rewarmed for 10 minutes, fixed with fixation solution for 15 minutes, and digested with proteinase K at 37℃ for 10 minutes. Sections were then hybridized with target probes 1 for COL1A1 and CCL21 at 40°C overnight (Table S10). After washed with 2×, 1×, 0.5×and 0.1×SSC for 5 minutes respectively, sections were hybridized with target probe 2 at 40°C for 45 min and washed with 2×, 1×, 0.5×and 0.1×SSC for 5 minutes again. Sections were then hybridized with fluorescence signal probe at 37°C for 3 hours and washed with 2×, 1×, 0.5×and 0.1×SSC for 5 minutes. Sections were then counterstained and mounted with Fluoroshield with DAPI (GeneTex, GTX30920). Images were finally acquired using ApoTome.2 microscope (Zeiss).

**Western blot**

Cells were lysed on ice with 200 μL RIPA buffer for 30 minutes. The lysate was collected into 1.5 mL tubes, centrifuged at 12,000 rpm for 15 minutes at 4°C and the supernatant was retained. Protein concentration was measured using the BCA method. 5× loading buffer were added, mixed, and heated at 100°C for 10 minutes to denature the proteins. After cooling, samples were loaded onto gels and separated by SDS-PAGE: 80 V for concentration gel and 120 V for separation gel. After transfer with 300mA for 90 minutes, the PVDF membrane was blocked with 5% non-fat milk for 2 hours, incubated with primary antibody for α-SMA (abcam, ab32575, 1:2000), Collagen I (Proteintech, 66761, 1:1000), Collagen III (Proteintech, 22734, 1:5000), Periostin (Proteintech, 66491, 1:5000), SOX9 (abcam, ab185966, 1:2000), and beta-actin (Proteintech, 66009, 1:10000) overnight at 4°C, and incubated with secondary antibody after membrane washing at room temperature for 1.5 h. Membranes were washed with TBST and detected using ECL.

**Supplementary figures**

**Fig. S1** Single cell transcriptome visualization of human RCT across samples. **a** Diagram of rat supraspinatus tendon rupture model. **b** Histological imaging of H&E and Masson staining in different time points of rat RCT model (3dpi, 14dpi and 60dpi for acute, subacute and chronic stages, respectively). **c** UMAP embedding of 87730 cells from 9 RCT samples annotated by samples with different injury times. **d** Violin plot showing nCount and nFeature values in different sample; **e** UMAP embedding showing expression of PTPRC, CD14, COL1A1 and COL1A2; **f** Proportion of major cell types in different samples.

**Fig. S2** Stemness, senescence and related SCENIC analysis of tenocytes**. a** UMAP embedding showing distributions of CytoTRACE2 and SID scores in tenocyte. **b** Violin plot showing SID score in different stages of total tenocytes. **c** Correlation between senescence score and MMP2 expression in tenocytes. **d** Heatmap showing specific activated regulons of different tenocyte subclusters. **e** Regulons significantly correlated with pseudotime.

**Fig. S3** Characteristics of smooth muscle cells (SMC) subclusters. **a** UMAP embedding of SMC annotated by stages and subclusters. **b** Dot heatmap showing significant upregulated genes of each subcluster. **c** Proportion of SMC subclusters in different RCT stages. **d** Boxplot showing expression of CCL2, POSTN, MMP2, CXCL12, COL1A1, THBS4 in different stages of each subcluster. **e** Heatmap showing activities of ECM-related pathways across SMC subclusters. **f** Trajectories of SMC annotated by pseudotime and subclusters. **g** Expression of COL1A1 and MMP2 along with pseudotime of SMC.

**Fig. S4** Profiles of immune cells participate in different RCT stages. **a** UMAP embedding of immune cells annotated by stages and cell types. **b** UMAP embedding showing common marker genes expression of each immune cell. **c** Fractions of immune cells in different RCT stages. **d** Multiplex immunofluorescence showing different immune cell infiltration in different RCT stages. **e** High-magnification view showing marker genes expression of different immune cells. **f** Measurement of population of different immune cells in different RCT stages (Acute, n=6; Subacute, n=6; Chronic, n=6). For bar plots, mean ± SD are shown, and P values were calculated by one-way ANOVA followed by Tukey’s multiple-comparison test. *P < 0.05, **P < 0.01, ***P < 0.001.

**Fig. S5** Expression and pathways characteristics of macrophages in different stages**. a** Volcano plot showing significantly expressed genes of macrophages in different stages. **b** Heatmap showing significantly upregulated pathways of macrophages in different stages. **c** Violin plot showing expressions of ECM-related genes of macrophages in different stages. **d** UMAP embedding showing expressions of COL1A1 and COL1A2 in macrophages.

**Fig. S6** Characteristics of lymphatic endothelial cells in different RCT stages. **a** Immunofluorescence showing α-SMA and CD31 expression in rat RCT model. **b, c** Measurement of CD31^+^ area and α-SMA intensity in CD31^+^ area in rat RCT model (n=4). **d** UMAP embedding of lymph-Endo annotated by stages. **e** Volcano plot showing differentially expressed genes of lymph-Endo compared to other endothelial cells. **f** Violin plots of LYVE1, CCL21, TFF3, and MMRN1 expression in endothelial subclusters. **g** Gene expression of CCL21 in lymph-Endo of different RCT stages. **h** FISH and immunofluorescence co-staining showing CCL21 expression in lymph-Endo. **i** Immunofluorescence showing Ki67 distribution in lymph-Endo. **j** Measurement of LYVE-1+ area in different RCT stages. **k** Measurement of Ki67+/LYVE-1+ percent in different RCT stages. **l** Measurement of CCL21 intensity in lymph-Endo in different RCT stages. (Acute, n=10; Subacute, n=13; Chronic, n=15). For bar plots, mean ± SD are shown, and P values were calculated by one-way ANOVA followed by Tukey’s multiple-comparison test. *P < 0.05, **P < 0.01, ***P < 0.001.

**Fig. S7** Intercellular communication and pro-fibrotic signaling profiles across all subclusters**. a** Communication network among all subclusters. **b** Interaction heatmap of LR pairs between macrophage subclusters and tenocyte subclusters. **c** Heatmap showing expression of SPP1 receptors in tenocyte subclusters. d Activities of pro-fibrotic signaling pathways (top) and expressions of pro-fibrotic factors (bottom) across all subclusters; the pie chart on the right of the bar plot showing the proportion of each factor expressed by each cell type. **i, j** Western blot analysis of collagen I, collagen III and periostin expression in primary rat tenocytes with OPN and/or TGF-β treatment (n=3). (mean ± SD are shown as error bar, *P < 0.05, **P < 0.01, ***P < 0.001)

**Fig. S8** Response characteristics of pro-fibrotic signaling. **a** Percentage bar plots showing response percentage in tenocyte, SMC, macrophage and endothelial subclusters. **b** Bar plots showing FRGs expression levels in response and no-response cells.

**Supplementary tables**

**Table S1. Clinical information of 38 RCT patients and 2 ACL tear patients.**

**Table S2. Marker gene sets of the major cell types.**

**Table S3. References of the eight fibrosis-related genes.**

**Table S4. Subcluster specific DEGs of tenocyte, SMC, myeloid and endothelial cells.**

**Table S5. GO enrichment** **analysis of tenocyte subclusters.**

**Table S6. GO enrichment analysis of SMC subclusters.**

**Table S7. GO enrichment analysis of macrophage subclusters.**

**Table S8. GO enrichment results and hub genes lists of 5 co-expression metaprograms of macrophages.**

**Table S9. GO enrichment analysis of endothelial subclusters.**

**Table S10. Sequences for FISH probes.**
