# Supplementary figures and images for: Single cell atlas decodes the molecular dynamics of scar repair after human rotator cuff tear
Source: Bone Res. 2026 Feb 5;14:17. doi: 10.1038/s41413-025-00501-5 (PMC12877062; doi:10.1038/s41413-025-00501-5)

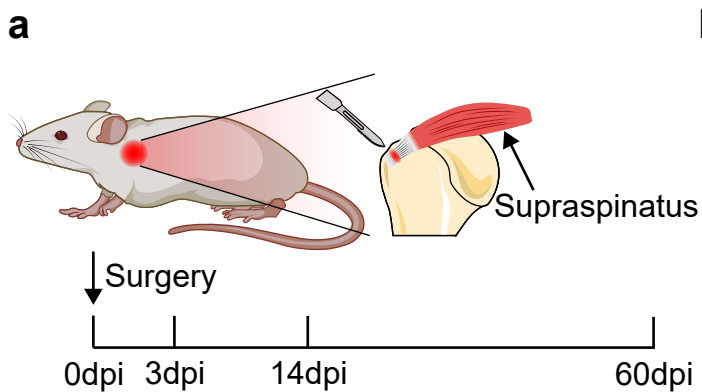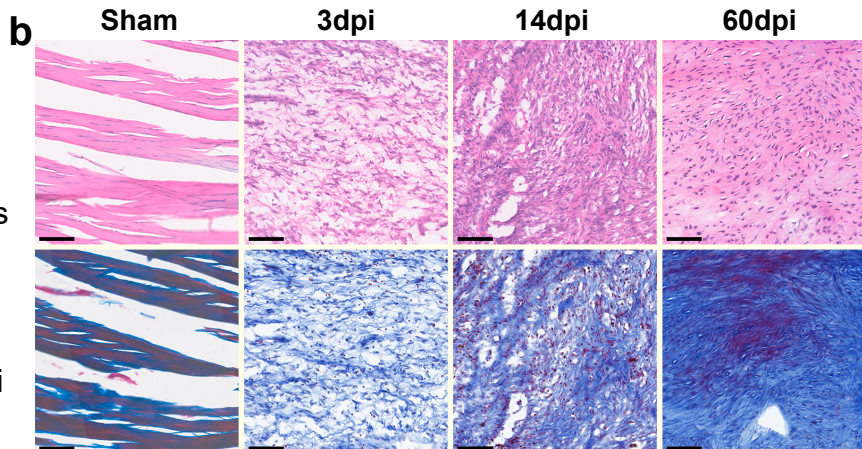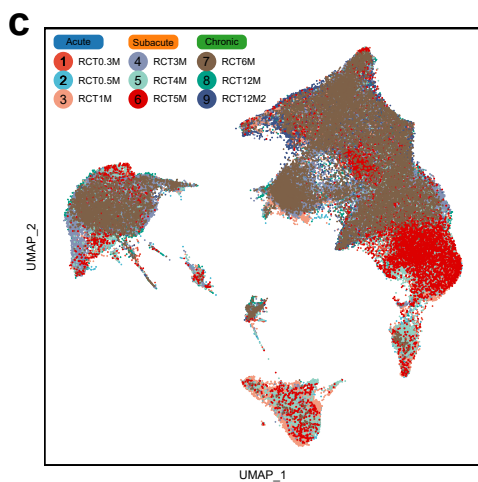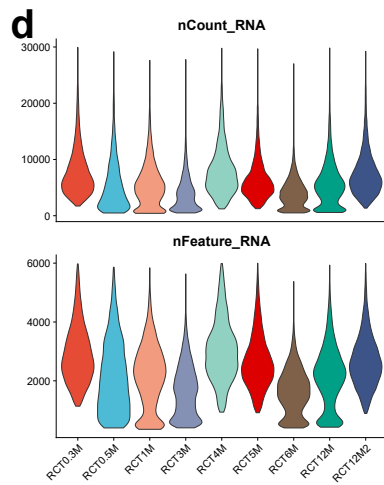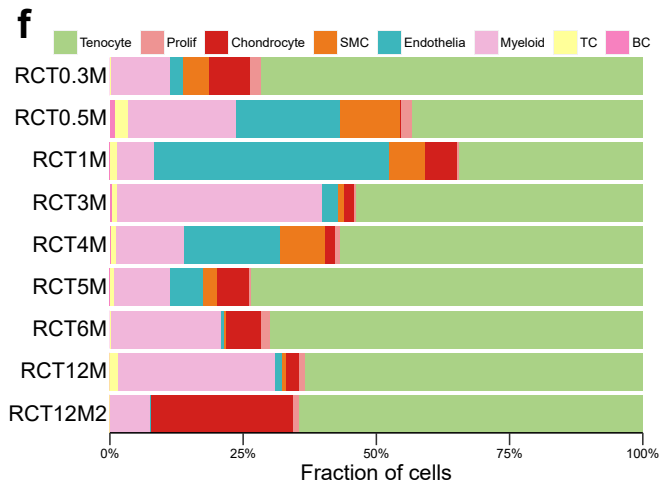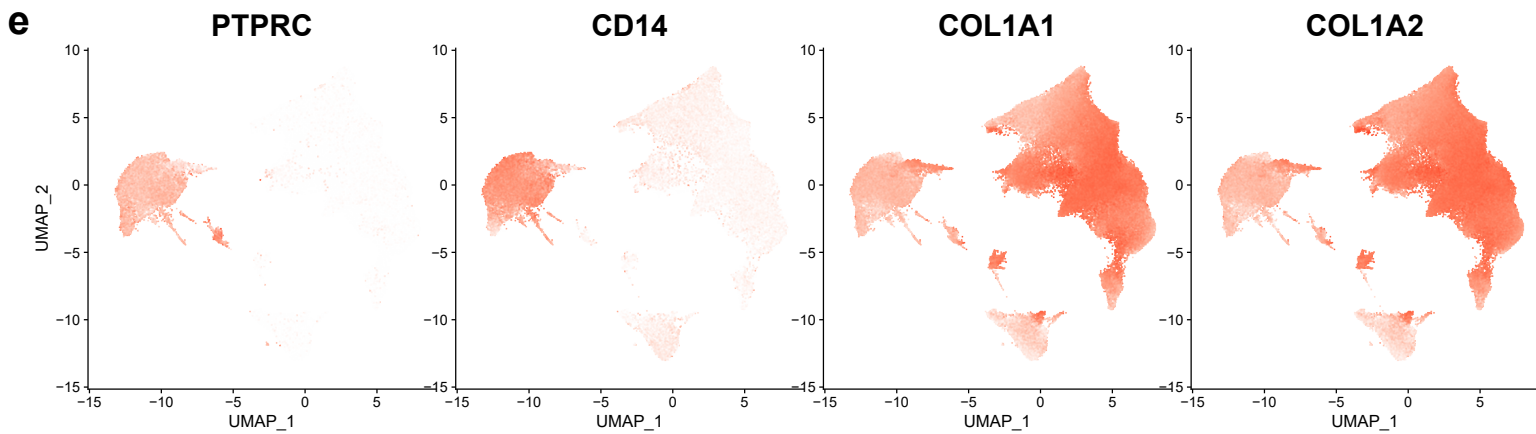

Supplement: Supplementary file 12 — Supplementary Figure S1 [file 41413_2025_501_MOESM12_ESM.pdf]

**a**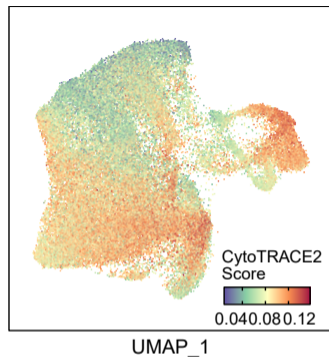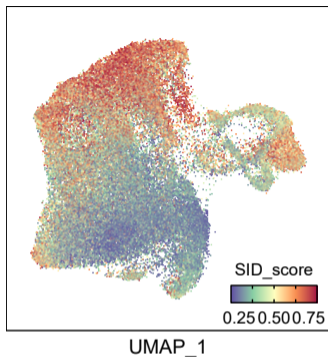**b**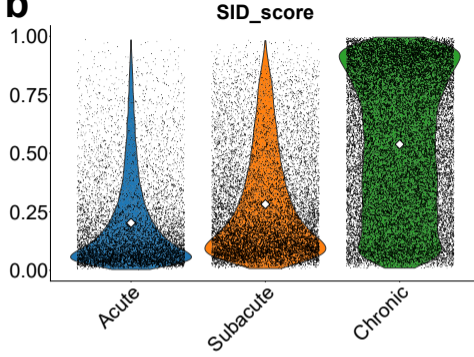**c**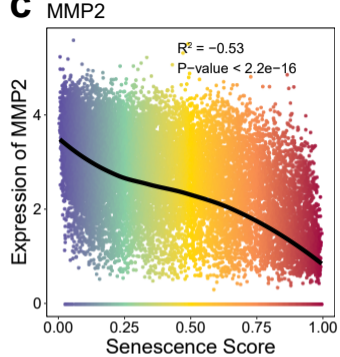**d**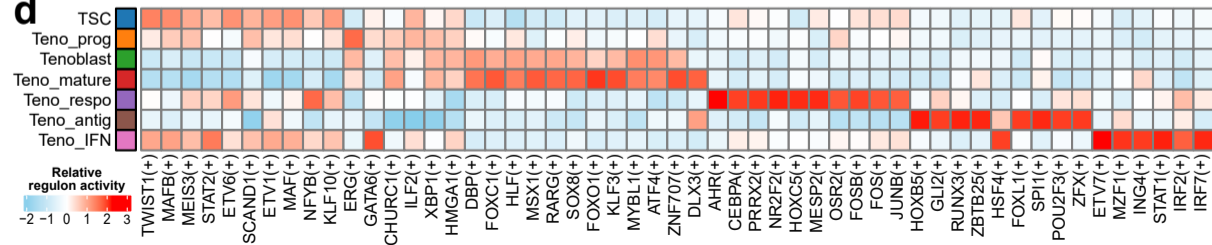**e**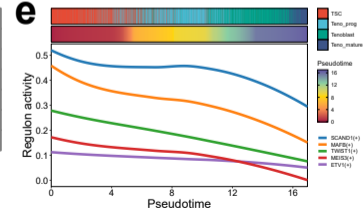

Supplement: Supplementary file 13 — Supplementary Figure S2 [file 41413_2025_501_MOESM13_ESM.pdf]

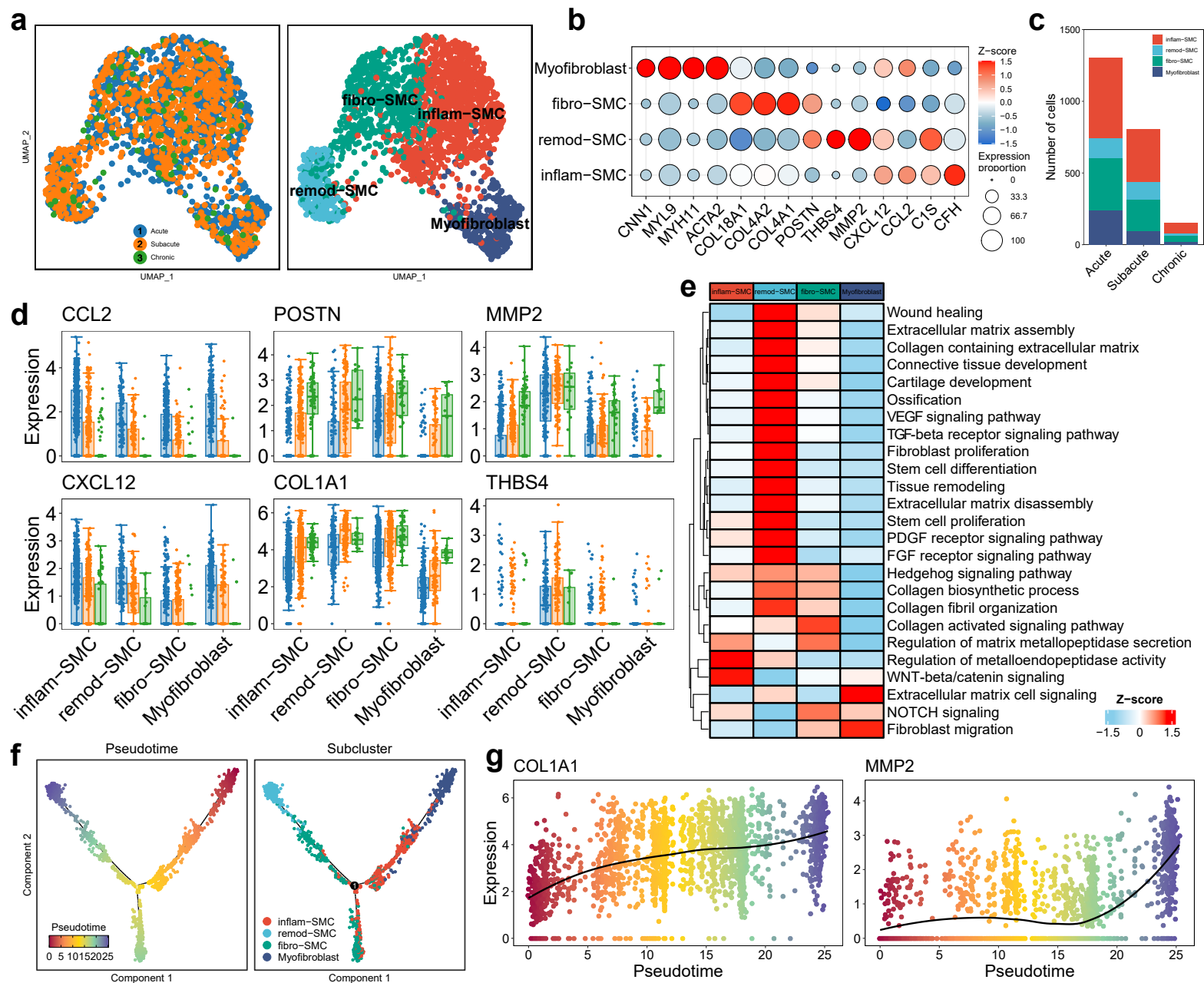

Supplement: Supplementary file 14 — Supplementary Figure S3 [file 41413_2025_501_MOESM14_ESM.pdf]

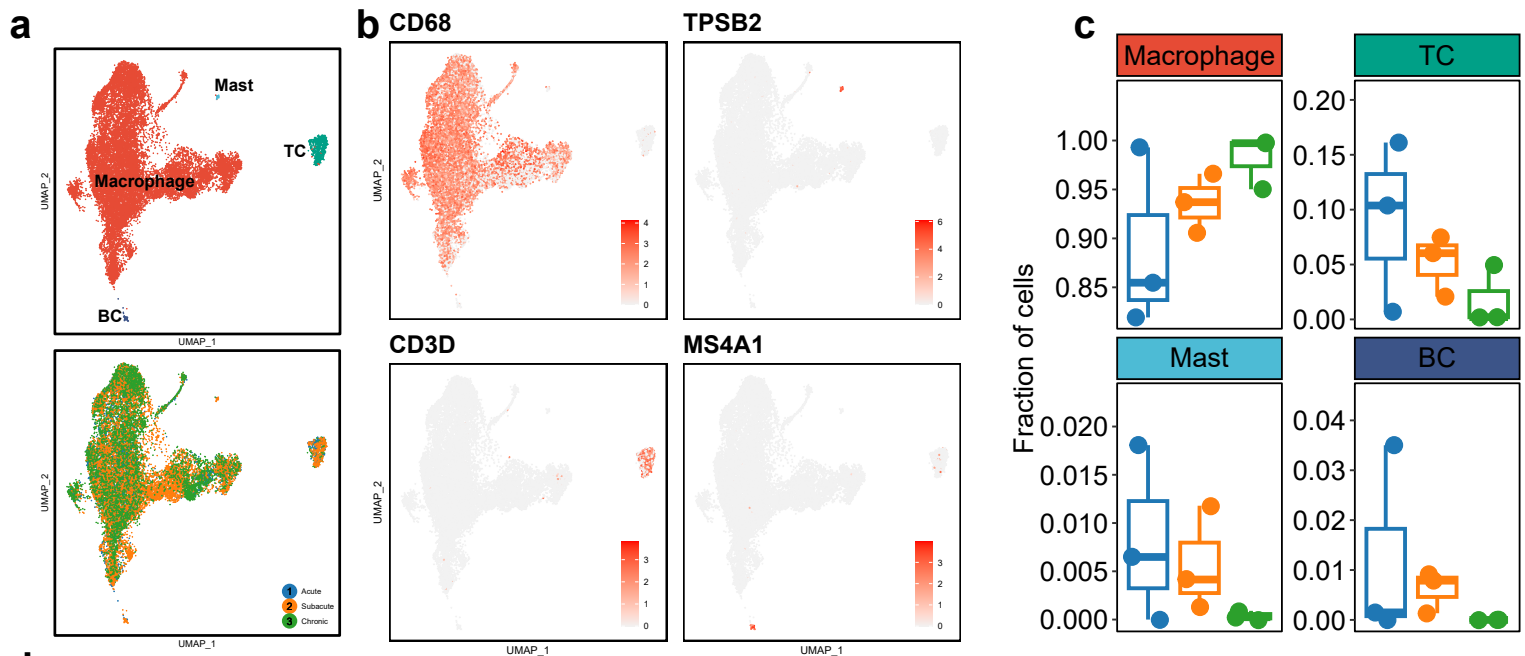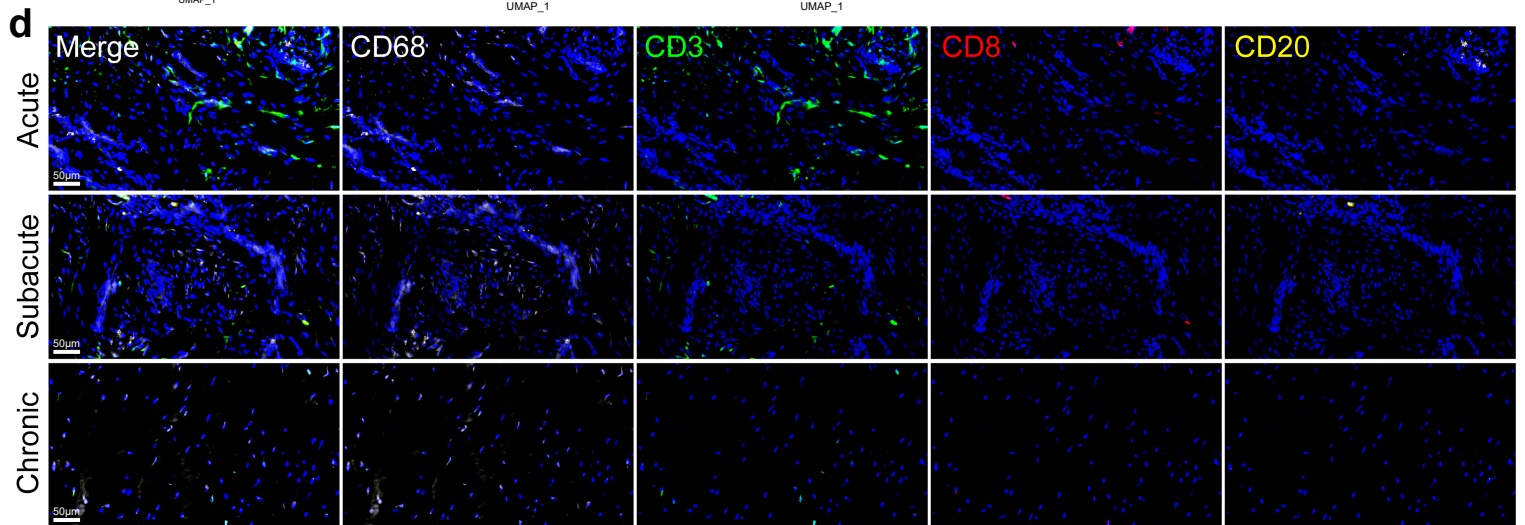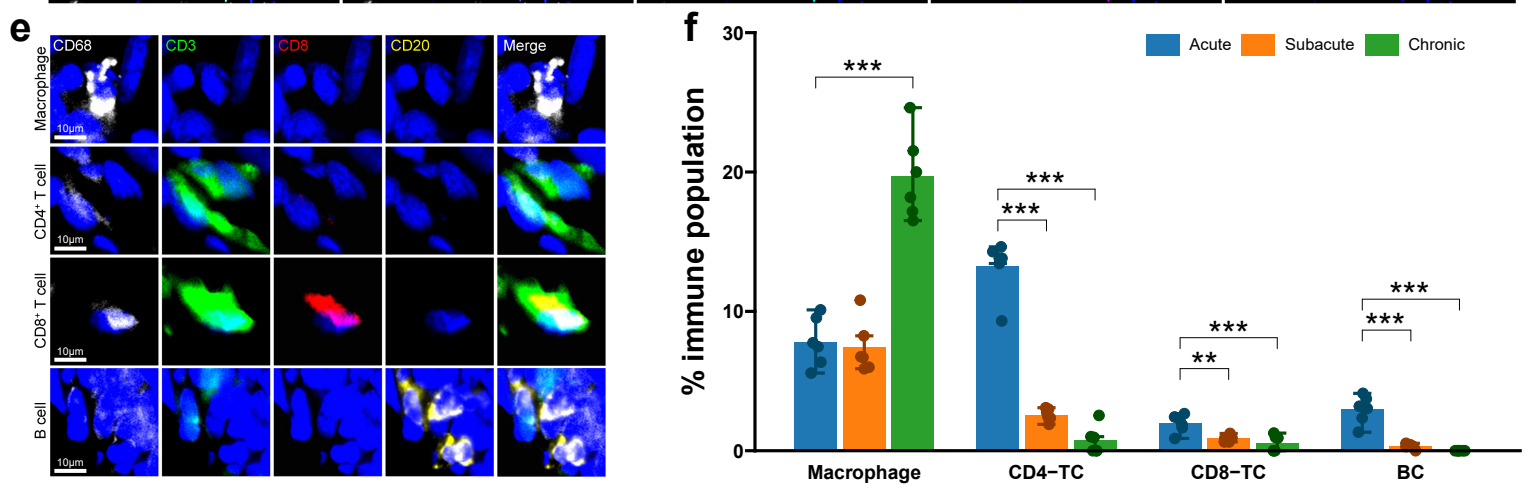

Supplement: Supplementary file 15 — Supplementary Figure S4 [file 41413_2025_501_MOESM15_ESM.pdf]

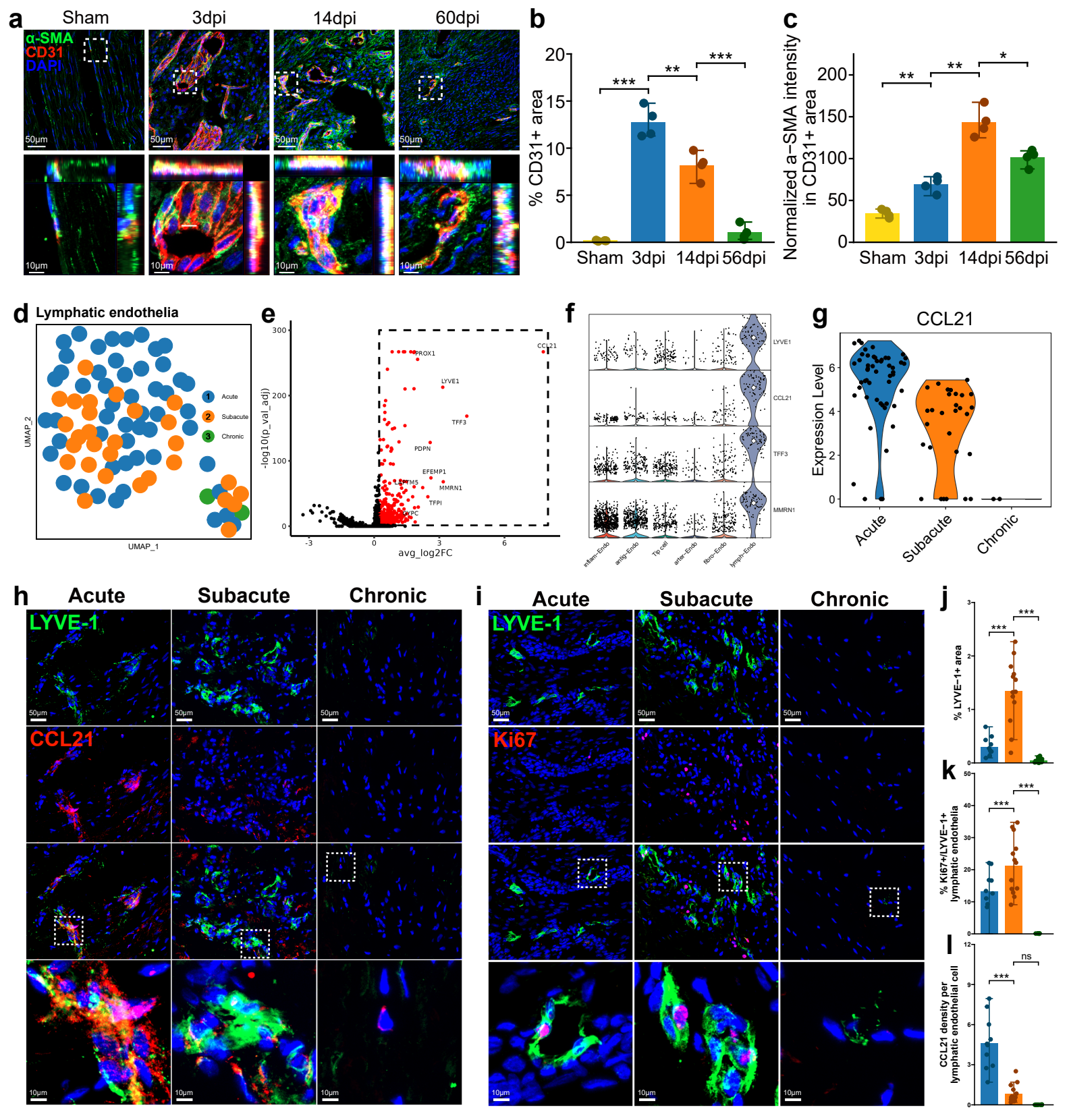

Supplement: Supplementary file 17 — Supplementary Figure S6 [file 41413_2025_501_MOESM17_ESM.pdf]

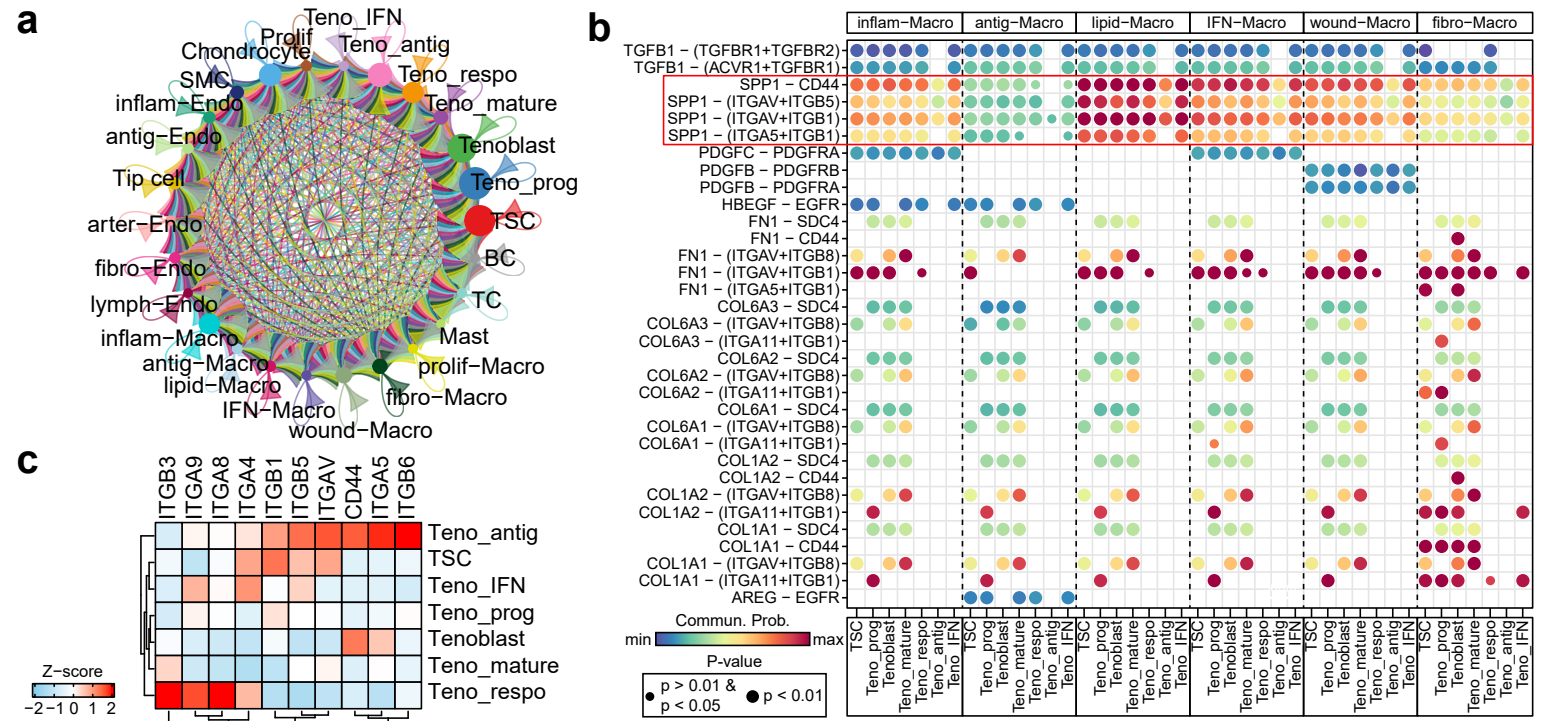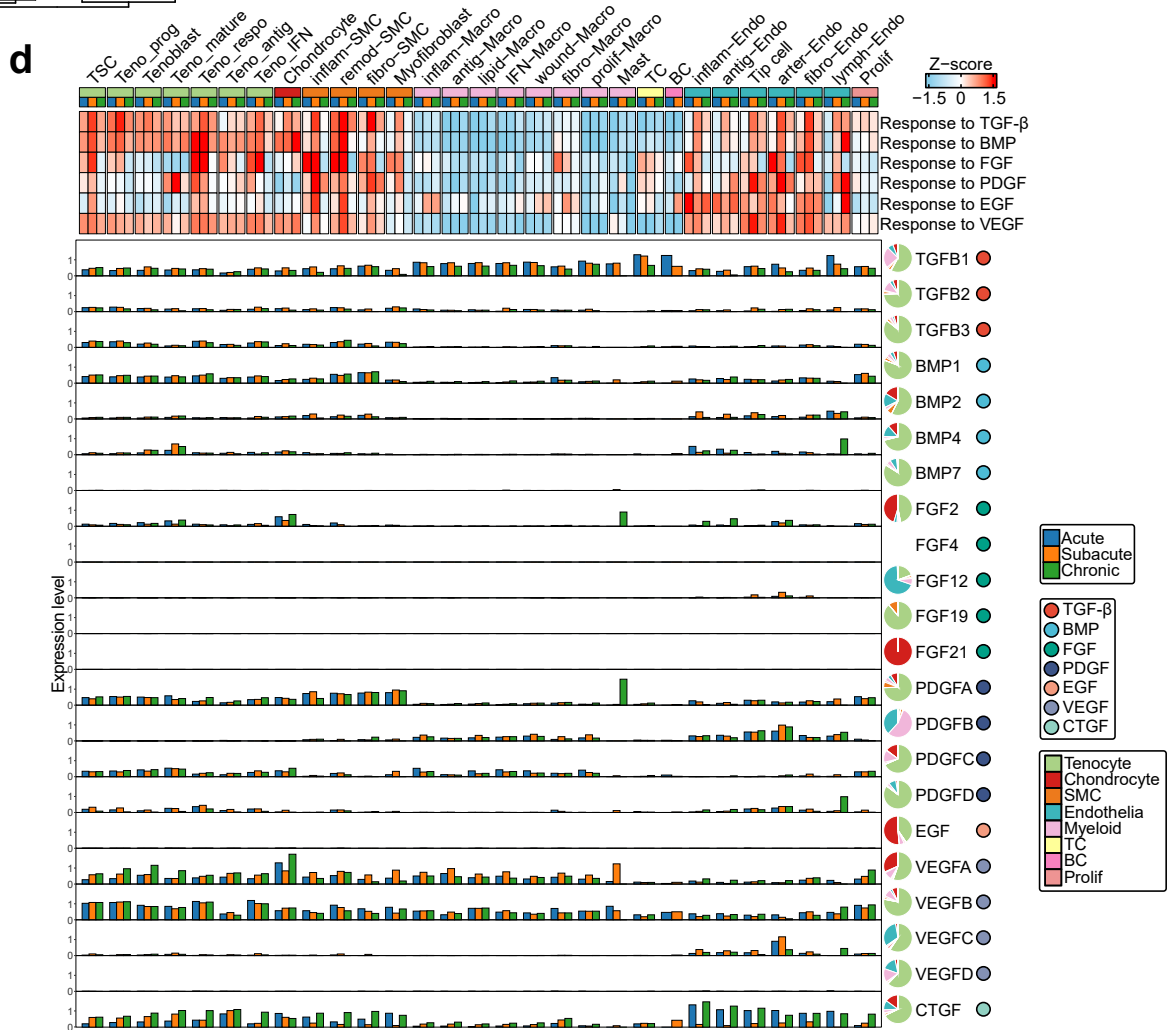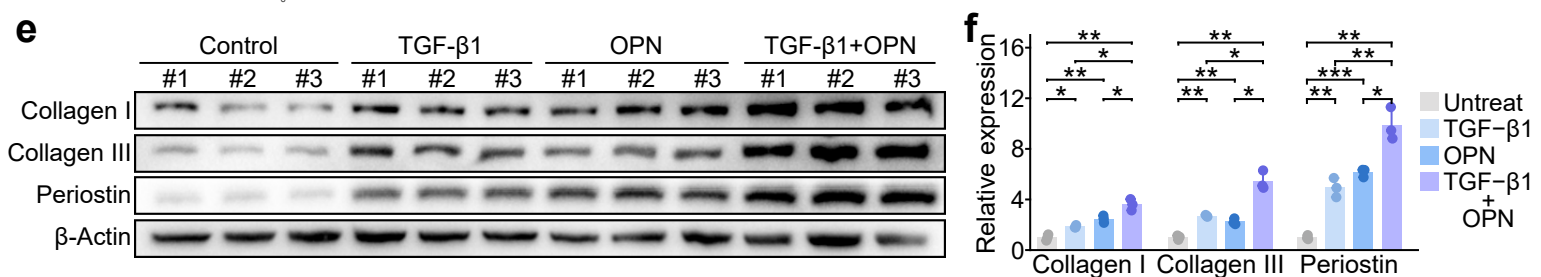

Supplement: Supplementary file 18 — Supplementary Figure S7 [file 41413_2025_501_MOESM18_ESM.pdf]

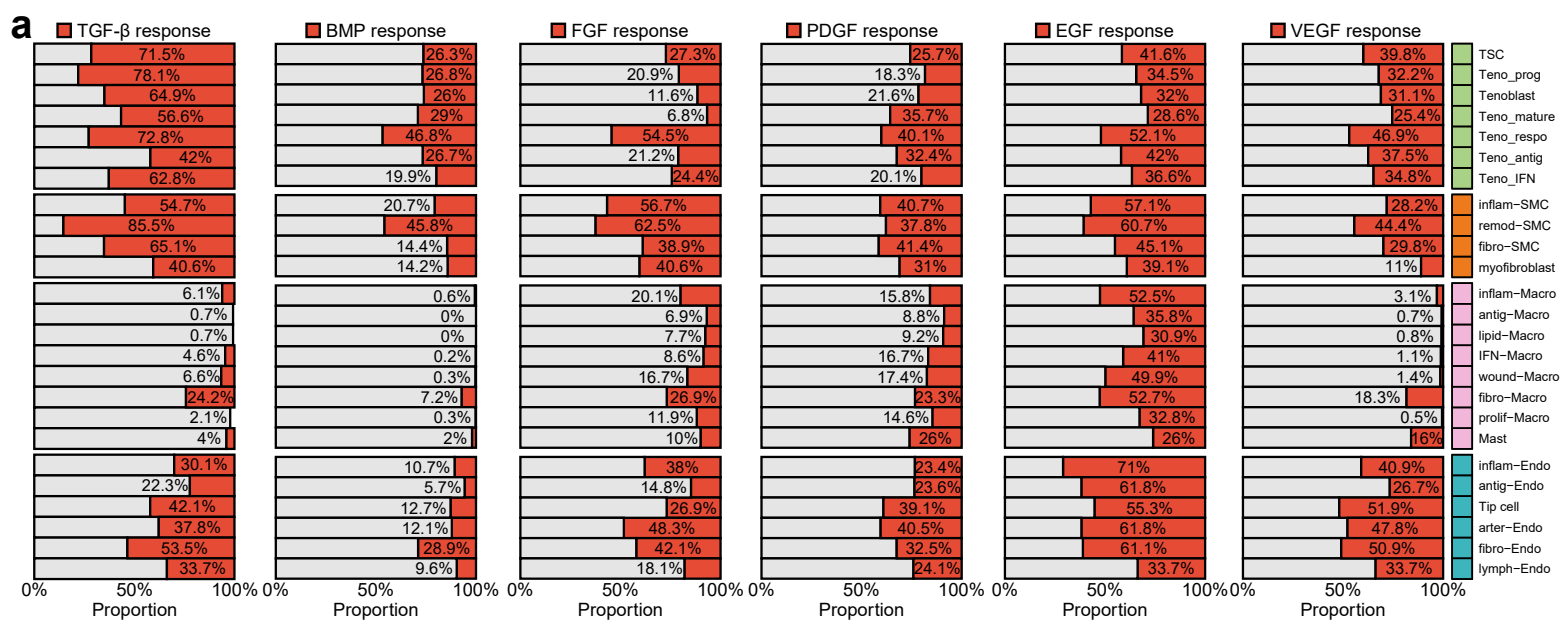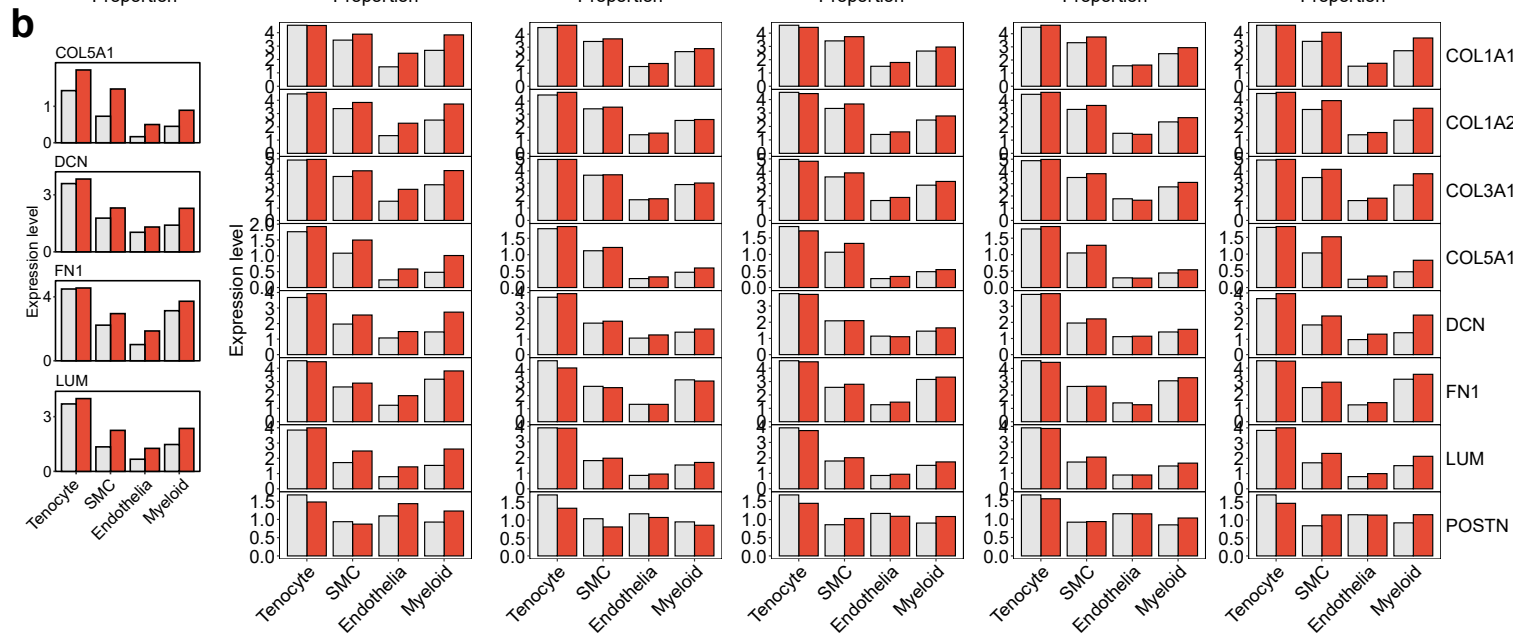

Supplement: Supplementary file 19 — Supplementary Figure S8 [file 41413_2025_501_MOESM19_ESM.pdf]
