# Supplementary material for: Single cell atlas decodes the molecular dynamics of scar repair after human rotator cuff tear
Source: Bone Res. 2026 Feb 5;14:17. doi: 10.1038/s41413-025-00501-5 (PMC12877062; doi:10.1038/s41413-025-00501-5)

Figure 6k SOX9 (57kDa)

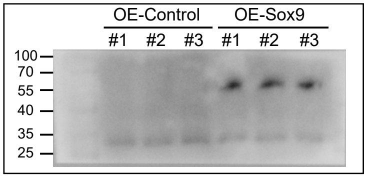

Figure 6k α-SMA (42kDa)

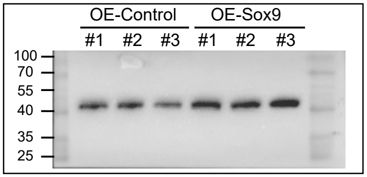

Figure 6k Collagen III (170kDa)

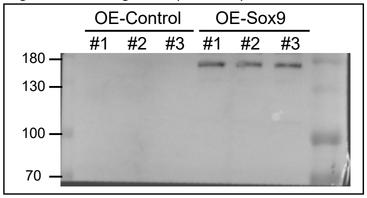

Figure 6k Periostin (93kDa)

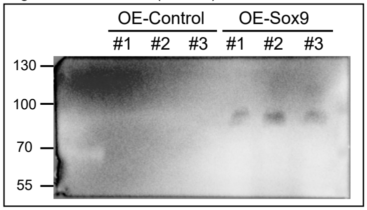

Figure 6k β-Actin (42kDa)

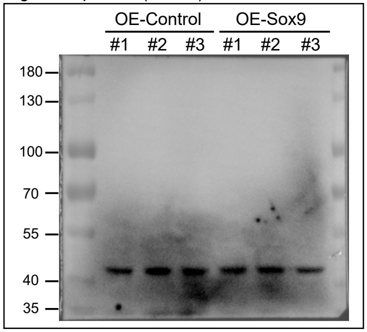

Figure 6k Collagen I (130-180kDa)

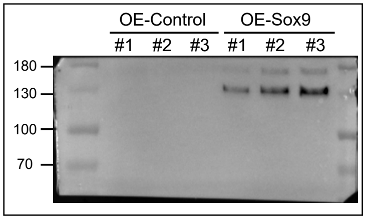

Figure 8g Collagen I (130-180kDa)

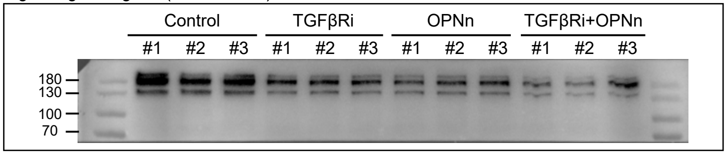

Figure 8g Collagen III (170kDa)

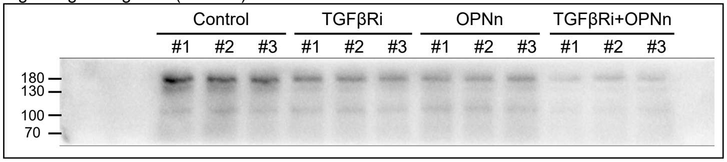

Figure 8g Periostin (93kDa)

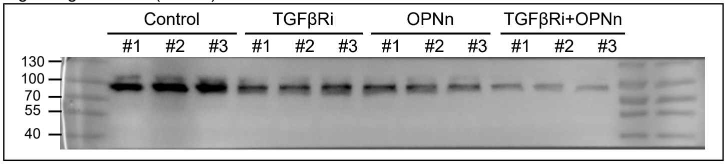

Figure S7e Collagen I (130-180kDa)

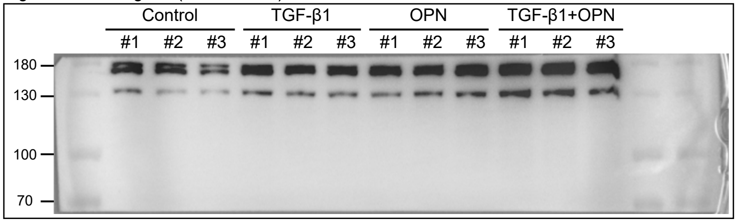

Figure S7e Collagen III (170kDa)

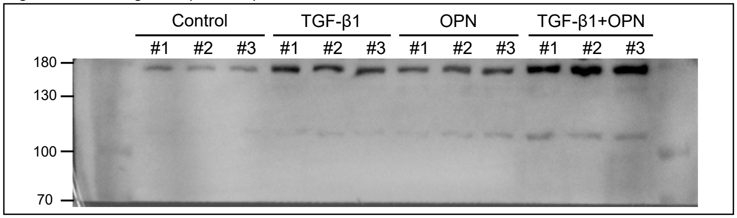

Figure S7e Periostin (93kDa)

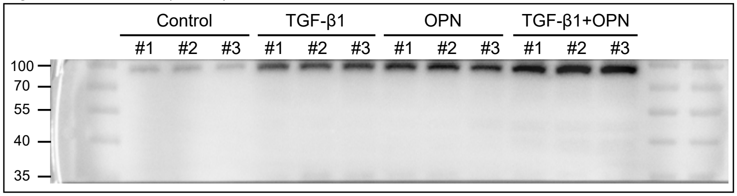

Figure S7e β-Actin (42kDa)

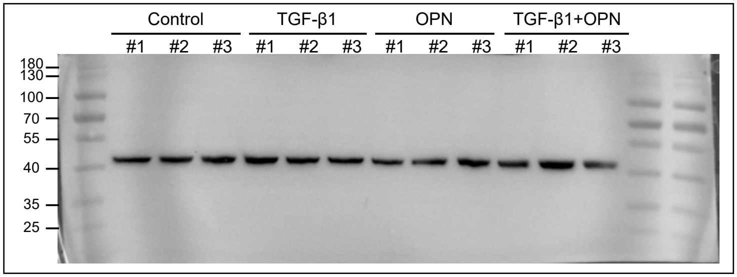

Figure 8g β-Actin (42kDa)

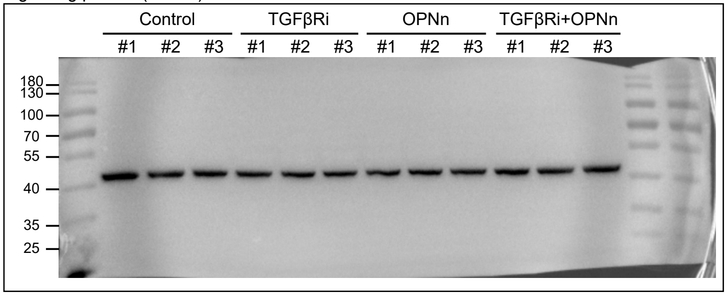

Supplement: Supplementary file 20 — Original WB images [file 41413_2025_501_MOESM20_ESM.pdf]
