# Supplementary material for: Single cell atlas decodes the molecular dynamics of scar repair after human rotator cuff tear
Source: Bone Res. 2026 Feb 5;14:17. doi: 10.1038/s41413-025-00501-5 (PMC12877062; doi:10.1038/s41413-025-00501-5)
Supplement: Supplementary file 16 — Supplementary Figure S5 [file 41413_2025_501_MOESM16_ESM.pdf]

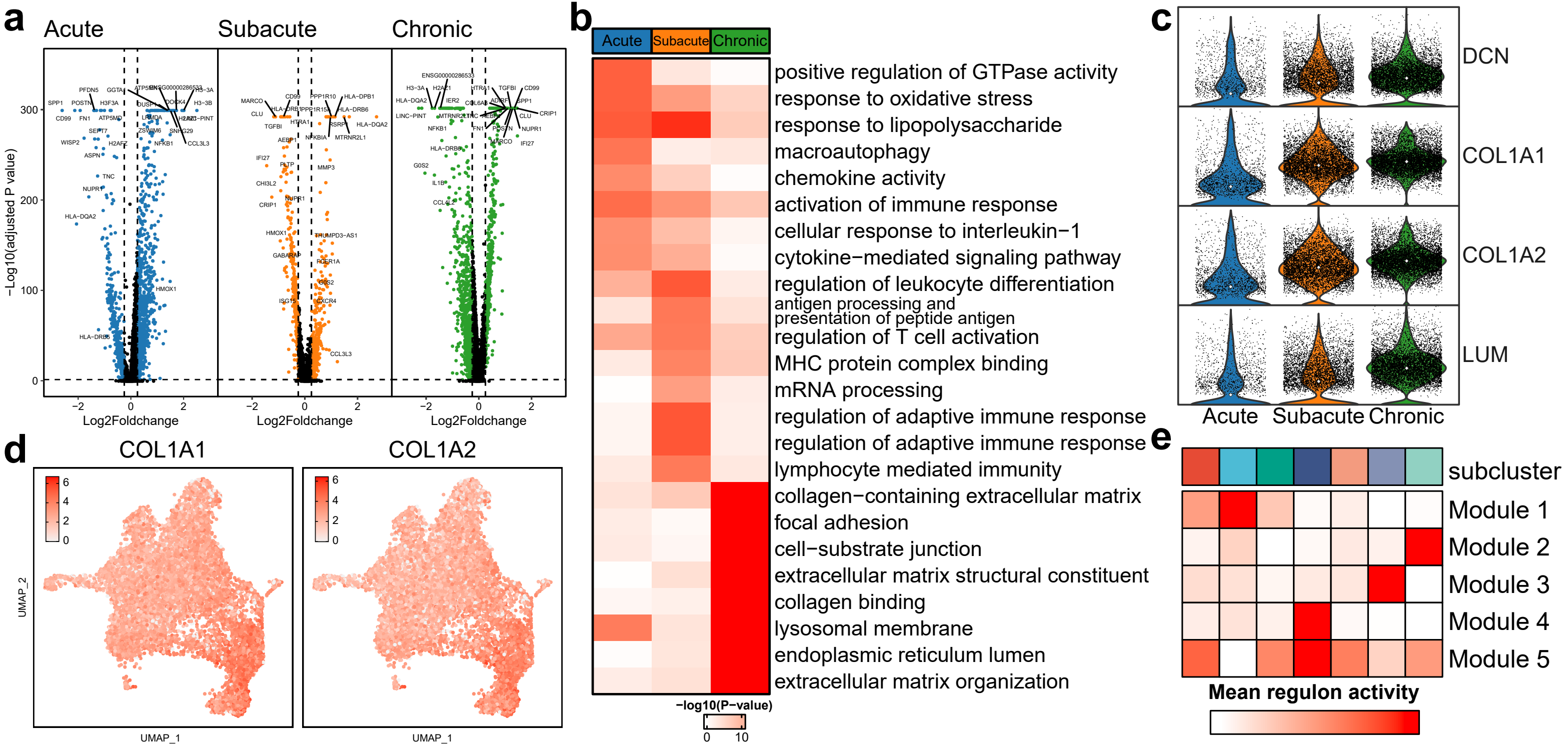

a
Acute
Subacute
Chronic
−Log10(adjusted P value)
Log2Foldchange
b
positive regulation of GTPase activity
response to oxidative stress
response to lipopolysaccharide
macroautophagy
chemokine activity
activation of immune response
cellular response to interleukin−1
cytokine−mediated signaling pathway
regulation of leukocyte differentiation
antigen processing and presentation of peptide antigen
regulation of T cell activation
MHC protein complex binding
mRNA processing
regulation of adaptive immune response
regulation of adaptive immune response
lymphocyte mediated immunity
collagen−containing extracellular matrix
focal adhesion
cell−substrate junction
extracellular matrix structural constituent
collagen binding
lysosomal membrane
endoplasmic reticulum lumen
extracellular matrix organization
−log10(P−value)
0
10
c
DCN
COL1A1
COL1A2
LUM
Acute
Subacute
Chronic
d
COL1A1
COL1A2
UMAP_1
UMAP_2
e
subcluster
Module 1
Module 2
Module 3
Module 4
Module 5
Mean regulon activity
